# Supplementary material for: Selective electrochemical reduction of nitric oxide to hydroxylamine by atomically dispersed iron catalyst
Source: Nat Commun. 2021 Mar 25;12:1856. doi: 10.1038/s41467-021-22147-7 (PMC7994811; doi:10.1038/s41467-021-22147-7)
Supplement: Supplementary file 1 — Supplementary Information [file 41467_2021_22147_MOESM1_ESM.pdf]

Supporting Information

Selective electrochemical reduction of nitric oxide to hydroxylamine by atomically dispersed iron catalyst

Dong Hyun Kim[1†], Stefan Ringe[2†], Haesol Kim[1], Sejun Kim[3], Bupmo Kim[4], Geunsu Bae[1], Hyung-Suk Oh[5], Frédéric Jaouen[6], Wooyul Kim[7]*, Hyungjun Kim[3]* and Chang Hyuck Choi[1]*

[1]*School of Materials Science and Engineering, Gwangju Institute of Science and Technology, Gwangju 61005, Republic of Korea.*

[2]*Department of Energy Science and Engineering, Daegu Gyeongbuk Institute of Science and Technology, Daegu 42988, Republic of Korea.*

[3]*Department of Chemistry, Korea Advanced Institute of Science and Technology, Daejeon 34141, Republic of Korea.*

[4]*Department of Chemical Engineering, Pohang University of Science and Technology, Pohang 37673, Republic of Korea.*

[5]*Clean Energy Research Center, Korea Institute of Science and Technology, Seoul 02792, Republic of Korea.*

[6]*ICGM, Université de Montpellier, CNRS, ENSCM, 34090 Montpellier, France.*

[7]*Department of Chemical and Biological Engineering, Sookmyung Women's University, Seoul 04310, Republic of Korea.*

[†]*These authors contributed equally to this work.*

**Correspondence to: Wooyul Kim (wkim@sookmyung.ac.kr), Hyungjun Kim (linus16@kaist.ac.kr), and Chang Hyuck Choi (chchoi@gist.ac.kr)*

CONTENTS

Supplementary Notes	....	2–10
Supplementary Figures	....	11–46
Supplementary Tables	....	47–51
Supplementary References	....	52–55

SUPPLEMENTARY NOTES

Supplementary Note 1. Physical characteristics of the Fe-N-C catalysts.

FeNC-dry-0.5

FeNC-dry-0.5 was prepared by pyrolysis of Fe^{II} acetate, 1,10-phenanthroline (phen), and Zn^{II} zeolitic imidazolate framework (ZIF-8) at 1,323 K. X-ray diffraction (XRD), Raman spectroscopy, transmission electron microscopy (TEM) show successful carbonisation of the precursor mixture after the pyrolysis (Supplementary Fig. 1a–c), and X-ray photoelectron spectroscopy (XPS) reveals introduction of N-functionalities into the carbon substrate (Supplementary Fig. 2a). ^{57}Fe Mössbauer spectroscopy shows only the doublets D1 and D2 (i.e., isolated FeN_xC_y moieties) without any discernable signals from the crystalline Fe particles (Supplementary Fig. 3a and Supplementary Table 1), indicating a sole presence of the atomically dispersed FeN_xC_y moieties in the prepared catalyst. This observation was also corroborated by extended X-ray absorption fine structure (EXAFS) with a strong backscattering signal of Fe-N(O) interaction without Fe-Fe interaction (Supplementary Fig. 4). Inductively coupled plasma mass spectrometry (ICP-MS) analysis indicated that the Fe content of FeNC-dry-0.5 is ca. 1.5 wt%, from which a considerable amount is on the catalyst surface as confirmed by a surface-specific nitrite stripping method (Supplementary Fig. 5).

FeNC-dry-1 and FeNC-wet-1

FeNC-dry-1 and FeNC-wet-1 catalysts were prepared as FeNC-dry-0.5 but with a duplicated Fe content. ICP-MS revealed that total Fe contents of FeNC-dry-1 and FeNC-wet-1 are ca. 3.0 and 3.4 wt%, respectively. XPS showed similar N-doping level and components for all the catalysts (Supplementary Fig. 2). Meanwhile, ^{57}Fe Mössbauer spectroscopy and EXAFS results confirmed the coexistence of Fe particles as well as FeN_xC_y moieties in the FeNC-dry-1 and FeNC-wet-1 (Supplementary Figs. 3–4 and Supplementary Table 1). Fe-N-C catalysts with high (ca. 1.2 wt.%, FeNC-wet-1) and low (ca. 0.2 wt%, FeNC-dry-1) contents of Fe particles were thus identified (Supplementary Fig. 3d), of which most were protected by graphite layers as confirmed by TEM images (Supplementary Fig. 1).

Supplementary Note 2. Estimation of NO concentration in a NO-saturated 0.1 M $HClO_4$ electrolyte and prediction of the number of electrons transferred at the 2nd reduction region.

NO concentration in a NO-saturated 0.1 M $HClO_4$ electrolyte was calculated from a nitric oxide reduction reaction (NORR) polarisation curve of polycrystalline Pt electrode collected at a 1,600 rpm rotation speed (Supplementary Fig. 6b). The measured diffusion-limited current density (j_d) at 0.4–0.25 V *vs.* reversible hydrogen electrode (RHE) was ca. 1.8 mA cm^{-2}. According to the Levich equation (Eq. 1), the j_d can be expressed in a term of NO concentration (C_{NO}) in the electrolyte.

$$j_d = 0.62nFD^{2/3}\omega^{1/2}\upsilon^{-1/6}C_{NO} \qquad \text{(Eq. 1)}$$

where n is the number of electrons transferred, F is the Faraday constant (96,485 C mol^{-1}), D is the diffusion coefficient of NO (2.5 × 10^{-5} cm^2 s^{-1}), ω is the rotation speed (rad s^{-1}), and υ is the kinematic viscosity of solution (8.5 × 10^{-3} cm^2 s^{-1}).[1] Because NORR on Pt surface produces N_2O at the diffusion-controlled potential region (n = 1),[1-4] the C_{NO} can be derived from the equation and the estimated value is 1.3 mM. This value is in accordance with that reported in literature (C_{NO} = 1.4 mM),[1] supporting again the selective N_2O production on Pt. Therefore, the Levich equation predicts that n value at the 2nd reduction region of NORR on FeNC-dry-0.5 is approximately 2.9 (j_d (@ the 2nd reduction region) / j_d (@ NO-to-N_2O conversion) = 5.2 / 1.8), inferring that three-electron transfer reaction (i.e., NO-to-NH_2OH conversion) predominantly occurs on FeNC-dry-0.5 at the 2nd reduction region.

Supplementary Note 3. Rotating ring disk electrode (RRDE) collection efficiency estimated with an assumption of selective NO-to-NH_2OH conversion on FeNC-dry-0.5.

The RRDE collection efficiency (N) was estimated from the voltammetric responses of Pt ring electrode during NORR on the FeNC-dry-0.5 disk electrode at a 1,600 rpm rotation speed in a NO-saturated 0.1 M $HClO_4$ electrolyte (Fig. 1d), a method suggested by Koper group.[2] The disk electrode was polarised either at 0.05 V_{RHE} or open circuit potential (OCP), a condition that FeNC-dry-0.5 catalyses NORR at the 2nd reduction region (Fig. 1a) or NORR does not occur, respectively. When NO was reduced on the disk at 0.05 V_{RHE} (i_{disk} = ca. 0.57 mA), decrease in Pt ring current was observed (Δi_{ring} = ca. 0.08 mA). Note that NORR follows one-electron pathway on Pt electrode at a potential above 0.25 V_{RHE}, producing N_2O as a final product. Because the current on the Pt ring electrode is proportional to the NO concentration at the ring electrode, which is the product of N and NO concentration at the disk electrode, the decrease in the ring current can be expressed as following equation (Eq. 2).

$$\begin{aligned}\Delta i_{ring} &= i_{ring}\,(@\,E_{disk} = \mathrm{OCP}) - i_{ring}\,(@\,E_{disk} = 0.05\ \mathrm{V_{RHE}}) \\ &\propto \text{1-electron} \times N \times C_{NO@OCP} - \text{1-electron} \times N \times C_{NO@0.05VRHE} \\ &\propto N \times (C_{NO@OCP} - C_{NO@0.05VRHE})\end{aligned} \quad \text{(Eq. 2)}$$

where $C_{NO@OCP}$ and $C_{NO@0.05VRHE}$ are the NO concentration at the disk electrode polarised at OCP and 0.05 V_{RHE}, respectively. Therefore, '$C_{NO@OCP} - C_{NO@0.05VRHE}$' is the NO concentration consumed at the disk electrode during the NORR at 0.05 V_{RHE}, which is proportional to the current on the FeNC-dry-0.5 disk electrode (Eq. 3).

$$i_{disk} \propto n \times (C_{NO@OCP} - C_{NO@0.05VRHE}) \quad \text{(Eq. 3)}$$

Accordingly, their proportionality derives the N value as a function of n (Eq. 4).

$$\Delta i_{ring} : i_{disk} = N \times (C_{NO@OCP} - C_{NO@0.05VRHE}) : n \times (C_{NO@OCP} - C_{NO@0.05VRHE})$$

$$N = n \times \Delta i_{ring} / i_{disk} \quad \text{(Eq. 4)}$$

Assuming that NORR on FeNC-dry-0.5 selectively produces NH_2OH at 0.05 V_{RHE} (i.e., $n = 3$), the N was estimated to ca. 0.42, which is in great agreement with the calibration value (Supplementary Fig. 10b). Therefore, this RRDE study confirms that FeNC-dry-0.5 mainly catalyses NO-to-NH_2OH conversion at the 2nd reduction region. This study further indicates that N_2O and NH_3 are not main NORR products on FeNC-dry-0.5 since these assumptions lead to unrealistically low and high N values of 0.14 and 0.71, respectively.

Supplementary Note 4. Detailed computational methods

Density functional theory (DFT) calculations

DFT calculations of reaction energetics were carried out with a periodic plane-wave implementation and ultra-soft pseudo-potentials using QUANTUM ESPRESSO version 6.1 interfaced with the atomistic simulation environment (ASE).[5,6] We applied ultra-soft pseudopotentials and the revised Perdew-Burke-Ernzerhof functional (RPBE).[7] Spin-polarised calculations were performed for which we determined optimally converged plane-wave and density cutoffs of 700 and 7,000 eV, respectively, and used a Fermi-level smearing width of 0.1 eV. Magnetic momenta were automatically optimised by QUANTUM ESPRESSO to minimise the total energy and are shown in Supplementary Table 2.

First, the 2D unit cell area of the Fe-N_4/graphene system was optimised by the Broyden-Fletcher–Goldfarb-Shanno (BFGS) line search algorithm. Subsequently, adsorption energies were calculated. For this, we used a 20 Å separation of the surface slabs, and 3 × 3 × 1 Monkhorst-Pack k-point grids.[8] The self-consistent continuum solvation (SCCS) implicit solvation model as implemented in the Environ QUANTUM ESPRESSO module was used to model the presence of implicit water. The 'fitg03' (in *a.u.*: $\rho_{\text{min}} = 0.0001$, $\rho_{\text{max}} = 0.005$) solvation parameter set is the default set that has been optimised for neutral molecule solvation energies.[9] The bulk dielectric permittivity was set to $\varepsilon_b = 6\varepsilon_0$ (vacuum permittivity ε_0) that has been rationalised in previous studies from the highly constrained water that has been observed at various metal surfaces.[10-16] Cavitation and repulsive energy terms are included by introducing an energy term proportional to the cavity surface area as described in the previous literature,[9] and we here apply the parameter $(\alpha + \gamma) = 11.5$ dyn cm^{-1} from the fitg03 parameter set. Dispersive solute-solvent interactions are ignored since they depend on the cavity volume which is an ill-defined property in surface slab calculations. The surface charge density was modulated in order to simulate the response of adsorbate free energies to the presence of an electric double layer field. A planar counter charge was placed at a distance of 5.5 Å over the graphene layer to neutralise the simulation cell. Previous studies showed that the choice of this distance does not affect the obtained energetics.[16] A parabolic correction was applied in the environ calculations to decouple the electrostatic interaction between the periodically repeated slabs.

Then the different systems were relaxed (all atoms were relaxed, no constrained atoms)

under the presence of varying surface charge density using the BFGS line search algorithm until force components were less than 0.03 eV Å^{-1}. Supplementary Fig. 15 shows the resulting optimised geometries, and Supplementary Table 2 all adsorption energies. The N_2O_2 adsorbate could only be stabilised with at least −0.4 e surface charge corresponding to around −4 µC cm^{-2}, at smaller charges adsorbed N_2O_2 dissociated into adsorbed NO and dissolved $NO_{(aq)}$. We therefore extrapolated the formation energy for smaller surface charge densities by performing single point calculations using the optimised geometry at −0.4 e surface charge/unit cell in line with previous studies.[16,17] Some systems exhibited particularly difficult convergence of the forces, although energies were converged in the meV region. We generated data at different surface charge density values, so that the energies could be interpolated by a parabolic function to get an analytic dependence of the adsorption energies on the surface charge density. Supplementary Fig. 16 shows all obtained surface charge dependencies.

The relaxed surface states were also re-calculated using the Vienna Abinito Simulation Package (VASP) as spin-polarised single point calculations using the RPBE+U and Heyd-Scuseria-Ernzerhof (HSE06) functional.[18,19] In the RPBE+U calculations, a value of U = 2 eV was applied to the l = 2 components of the Fe atom following previous studies which parametrised the U value to HSE06 calculations and experimental adsorption energies.[20] Core electrons of each element were treated with projected augmented wave (PAW) pseudopotential.[21,22] Considering the high computational costs of hybrid functional calculations, single gamma point and Monkhorst-pack 3 × 3 × 1 k-mesh in Brillouin zone sampling were used for reaction energy diagram and PDOS, respectively.

Finally, adsorbate vibrations were calculated using the finite difference scheme as implemented in ASE on the RPBE level under implicit solvation, but zero applied surface charge. From the vibrations, the zero-point energy (ZPE) and finite temperature corrections in the harmonic oscillator approximation were evaluated. In the case of the some adsorbates, they did not converge or were not stable at zero applied surface charge, so optimised structures at the smallest applied surface charge were used (Supplementary Fig. 15). No other energy corrections were applied, only the $H_2O_{(g)}$ energy level was referenced to liquid water by means of the solvation energy which however amounts to a negligible shift of the energy levels.[17]

Micro-kinetic model

A micro-kinetic model was created from the forward and backward rates of the following

reaction steps (where Fe^{II} refers to the whole catalyst):

(1) $NO_{(g)} + Fe^{II} \rightleftharpoons [Fe\text{-}NO\text{-}]^{\ddagger} \rightleftharpoons Fe^{II}\text{-}NO^{\delta-}$ (chemical)

(2) $Fe^{II}\text{-}NO^{\delta-} + H^{+}_{(aq)} + e^{-} \rightleftharpoons [Fe\text{-}NO\text{-}H]^{\ddagger} \rightleftharpoons Fe^{II}\text{-}NHO^{\delta-}$ (CHE)

(3) $Fe^{II}\text{-}NHO^{\delta-} + H^{+}_{(aq)} + e^{-} \rightleftharpoons [Fe\text{-}NHO\text{-}H]^{\ddagger} \rightleftharpoons Fe^{II}\text{-}NHOH^{\delta-}$ (CHE)

(4) $Fe^{II}\text{-}NHOH^{\delta-} + H^{+}_{(aq)} + e^{-} \rightleftharpoons [Fe\text{-}NHOH\text{-}H]^{\ddagger} \rightleftharpoons Fe^{II}\text{-}NH_2OH$ (CHE)

(5) $Fe^{II}\text{-}NH_2OH \rightleftharpoons [Fe\text{-}NH_2OH\text{-}]^{\ddagger} \rightleftharpoons Fe^{II} + NH_2OH_{(g)}$ (chemical)

(6) $Fe^{II}\text{-}NH_2OH + H^{+}_{(aq)} + e^{-} \rightleftharpoons [Fe\text{-}NH_2\text{-}H_2O]^{\ddagger} \rightleftharpoons Fe^{II}\text{-}NH_2^{\delta-} + H_2O_{(l)}$ (CHE)

(7) $Fe^{II}\text{-}NH_2^{\delta-} + H^{+}_{(aq)} + e^{-} \rightleftharpoons [Fe\text{-}NH_2\text{-}H]^{\ddagger} \rightleftharpoons Fe^{II}\text{-}NH_3$ (CHE)

(8) $Fe^{II}\text{-}NH_3 \rightleftharpoons [Fe\text{-}NH_3\text{-}]^{\ddagger} \rightleftharpoons Fe^{II} + NH_{3(g)}$ (chemical)

(9) $Fe^{II}\text{-}NO^{\delta-} + NO_{(g)} \rightleftharpoons [Fe\text{-}NO\text{-}NO]^{\ddagger} \rightleftharpoons Fe^{II}\text{-}N_2O_2^{\delta-}$ (chemical)

(10) $Fe^{II}\text{-}N_2O_2^{\delta-} + H^{+}_{(aq)} + e^{-} \rightleftharpoons [Fe\text{-}N_2O_2\text{-}H]^{\ddagger} \rightleftharpoons Fe^{II}\text{-}HN_2O_2^{\delta-}$ (CHE)

(11) $Fe^{II}\text{-}HN_2O_2^{\delta-} + H^{+}_{(aq)} + e^{-} \rightleftharpoons [Fe\text{-}HN_2O_2\text{-}H]^{\ddagger} \rightleftharpoons Fe^{II}\text{-}N_2O^{\delta-} + H_2O_{(l)}$ (CHE)

(12) $Fe^{II}\text{-}N_2O^{\delta-} \rightleftharpoons [Fe\text{-}N_2O\text{-}]^{\ddagger} \rightleftharpoons Fe^{II} + N_2O_{(g)}$ (chemical)

The pressure of NO gas was chosen as 0.5 bar (corresponding to the value that can be estimated from the actually measured NO concentration and Henry constant, Supplementary Note 2). Steps 1–5 are for NH_2OH formation, 1–4 and 6–7 for NH_3 formation, and 1 and 9–12 for N_2O formation. Reaction energetics for proton-coupled electron transfer (PCET) steps were evaluated based on the computational hydrogen electrode (CHE).[23] In addition, the impact of non-vanishing surface charge density/electric field was accounted for by first converting the actual electrode potential to a surface charge density. Assuming a constant double layer capacitance C_{dl} (ca. 20 μF cm^{-2} for graphene),[24,25] the surface charge density σ is generated according to:

$$\sigma = C_{dl}(E - E^{PZC}) \quad \text{(Eq. 5)}$$

where E is the applied electrode potential and E^{PZC} is the potential of zero charge (PZC) which we measured here to be 0 V *vs.* standard hydrogen electrode (SHE) (Supplementary Fig. 35). Then, the parabolic fits of the surface charge density dependence of Supplementary Fig. 16

were used to relate the potential to a field-stabilisation of the intermediate. This procedure was adapted from a previous publication to which the interested reader is referred to.[17]

We used the CATMAP simulation package to solve the coupled mean-field rate equations in the steady-state assumption.[26] Electrochemical barriers were estimated by referencing to the reversible potential of each electrochemical reaction step.[27] The rate constant for a particular elementary reaction step i is given as:

$$k_i(E) = A\ e^{-G_{a,i}(E)/RT} \quad \text{(Eq. 6)}$$

with the pre-exponential factor A, the applied voltage E, and the activation energy $G_{a,i}$. The activation energy can be expressed with reference to the reversible potential of the step as:

$$G_{\mathrm{a},i}(E) = G_{a,i}(E_i^0) + \beta \times F \times (E - E_i^0) \quad \text{(Eq. 7)}$$

where β (assumed to be 0.5) is the symmetric factor, F is the Faraday constant, and U_i^0 the reversible potential of the corresponding electrochemical reaction step. In the reversible potential model of Hansen *et al.*, $G_{a,i}(E_i^0)$ is assumed to be the same for all elementary reaction steps $G_{a,i}(E_i^0) = G_a(E^0)$. We used a value of 0.3 eV for this 'fixed' (potential-independent) barrier, but also tested a barrier of 0 which did not result in any qualitative changes in the polarisation curve. At zero fixed barrier, however, the N_2O pathway was found to be limited by the N_2O_2 formation step over the whole potential range (in contrast to the figure in the main paper showing some partial limitation at high overpotentials by a proton-electron transfer step).

For the remaining steps which do not involve the transfer of a proton-electron pair (NO adsorption, NO dimerisation, and the desorption reactions), a zero kinetic barrier was assumed. For NO adsorption and dimerisation, this assumption is valid, because the thermodynamic driving force is dominating over the relevant potential range with a strongly down-hill adsorption of NO and uphill formation of Fe^{II}-$N_2O_2^{\delta-}$. We did, however, also test a 0.5 eV barrier for NO adsorption, which we saw to only reduce the current densities slightly at the highest overpotentials (Supplementary Fig. 22). For the desorption all barriers were assumed to be zero, but a 0.5 eV barrier was tested for all 3 barriers to estimate the impact of the approximation. For NH_2OH adsorption, the introduction of a desorption barrier shifts the NH_2OH partial current downward and NH_2OH desorption can become limiting. This, however, would be inconsistent with the experimental pH trends, which is why we conclude that the

barrier must be smaller and less important. For NH_3, the desorption barrier was found to have no influence. For N_2O, the desorption barrier let to a decrease of all currents, however, outside the experimentally relevant potential range.

The active site density was needed to convert the turn over frequency provided by micro-kinetic modeling to a current density. We calculated it based on the experimental data of the FeNC-dry-0.5 catalyst and normalised to geometric area as:

$$\rho_{act} = \frac{\#\text{Fe active sites on electrode}}{\text{electrode geometric area (cm}^2)} = \frac{2.26 \cdot 10^{16} \text{ sites}}{1 \text{ cm}^2} = 2.26 \text{ sites/Å}^2$$

$$\#\text{Fe active sites on electrode} = \frac{\text{Fe active amount on the electrode (g)}}{\text{Fe atomic weight } \left(\frac{\text{g}}{\text{atom}}\right)}$$

$$= \frac{2.1 \text{ µg}}{9.27 \cdot 10^{-23} \text{g/atom}} = 2.26 \cdot 10^{16} \text{ sites}$$

$$\begin{aligned} &\text{Fe active amount on the electrode (g)} \\ &\quad = \text{Fe active per total Fe (\%)} \cdot \text{Total Fe wt (\%)} \\ &\quad \cdot \text{catalyst loading on electrode (µg/cm}^2) \cdot \text{electrode area (cm}^2) \\ &\quad = 0.2 \cdot 1.5 \text{ wt\%} \cdot 700 \text{ µg/cm}^2 \cdot 1 \text{ cm}^2 = 2.1 \text{ µg} \end{aligned}$$

Despite of our best efforts to estimate the active site density, it is extremely difficult to quantify how many active sites are actually exposed on the surface and able to participate in the catalytic reaction. Moreover, our Fe-N-C catalyst is a highly porous material, where a mass transport effect cannot be fully ignored even at the low overpotential regime. We thus found some quantitative discrepancy between theory and experiment (Supplementary Fig. 22d). Of particular, experimental current density showed an early leveling off behavior in comparison to the theoretical data, which can be attributed to the substantial mass transport effect. However, even with such approximations, our micro-kinetic model can correctly describe that N_2O is formed at lower and NH_2OH/NH_3 at higher overpotentials. Most importantly, it also described the experimentally observed inverse pH trends on an RHE scale by the rate-limiting steps and charge dependence, and also the key intermediates proposed from theory were successfully captured from our additional in situ IR experiments

Exchange-correlation functional dependence of the results

From the energy shifts in Supplementary Table 2, we first note that as expected the improved exchange description in both HSE06 and RPBE+U2 functional destabilises almost all adsorption states relative to the RPBE calculation. Later reaction intermediates are not critical as they are highly stabilised under the typically applied electrochemical potentials. However, the stronger destabilisation of the Fe^{II}-$NHO^{\delta-}$ state in the HSE06 calculations relative to the Fe^{II}-$NHOH^{\delta-}$ and Fe^{II}-NH_2OH states has a direct consequence on the rate-limiting step of NH_2OH production. Although the rate-limiting step in the HSE06 calculations becomes the Fe^{II}-$NO^{\delta-}$ to Fe^{II}-$NHO^{\delta-}$ step over almost the full potential range (Supplementary Fig. 19), while at the RPBE level different states were rate-limiting depending on the potential (Supplementary Fig. 17). This reduces the super-Nernstian shift, which, however, still is around 60 mV for NH_3 and about the same value for most potentials for NH_2OH (Supplementary Fig. 20). Notable is also the reduction of the Fe^{II}-$NO^{\delta-}$ coverage at low overpotentials due to destabilisation of that state. N_2O production, in contrast, is limited by the Fe^{II}-$N_2O_2{}^{\delta-}$ formation step in both RPBE and HSE06 calculations, so that no pH dependence results on an SHE scale. As a result, the HSE06 calculations qualitatively reproduce the pH trends with the RPBE calculations, yet, with different shift values and rate-limiting steps.

In contrast to the HSE06 functional, the RPBE+U2 functional over-destabilises the Fe^{II}-$NO^{\delta-}$ state relative to the following states in the NH_2OH production pathway. As shown in Supplementary Fig. 18, this results in a rate-limiting step from Fe^{II}-$NHO^{\delta-}$ to Fe^{II}-$NHOH^{\delta-}$ in the potential range from −0.4 to −0.2 V_{RHE} at pH 0 and Fe^{II}-$NHO^{\delta-}$ to mostly cover the surface in this region. This leads to a huge drop of the current with pH, much larger than expected from the experimental trends (Supplementary Fig. 20). Besides that, the Fe^{II}-$N_2O_2{}^{\delta-}$ intermediate is as destabilised as it is in the HSE06 calculation relative to the Fe^{II}-$NO^{\delta-}$ state. This makes the Fe^{II}-$N_2O_2{}^{\delta-}$ formation from Fe^{II}-$NO^{\delta-}$ feasible resulting in various steps being rate-limiting with Nernstian overpotential shifts and unrealistically high current densities (Supplementary Fig. 20). Considering the resulting pH dependence of N_2O formation on an SHE scale together with the obtained independence from experimental data, we conclude that the RPBE+U2 calculation is not an optimal choice for this system. It might require a more careful optimisation of the *U* parameter and maybe even application to orbitals of the often radical adsorbate states to improve this description.

SUPPLEMENTARY FIGURES

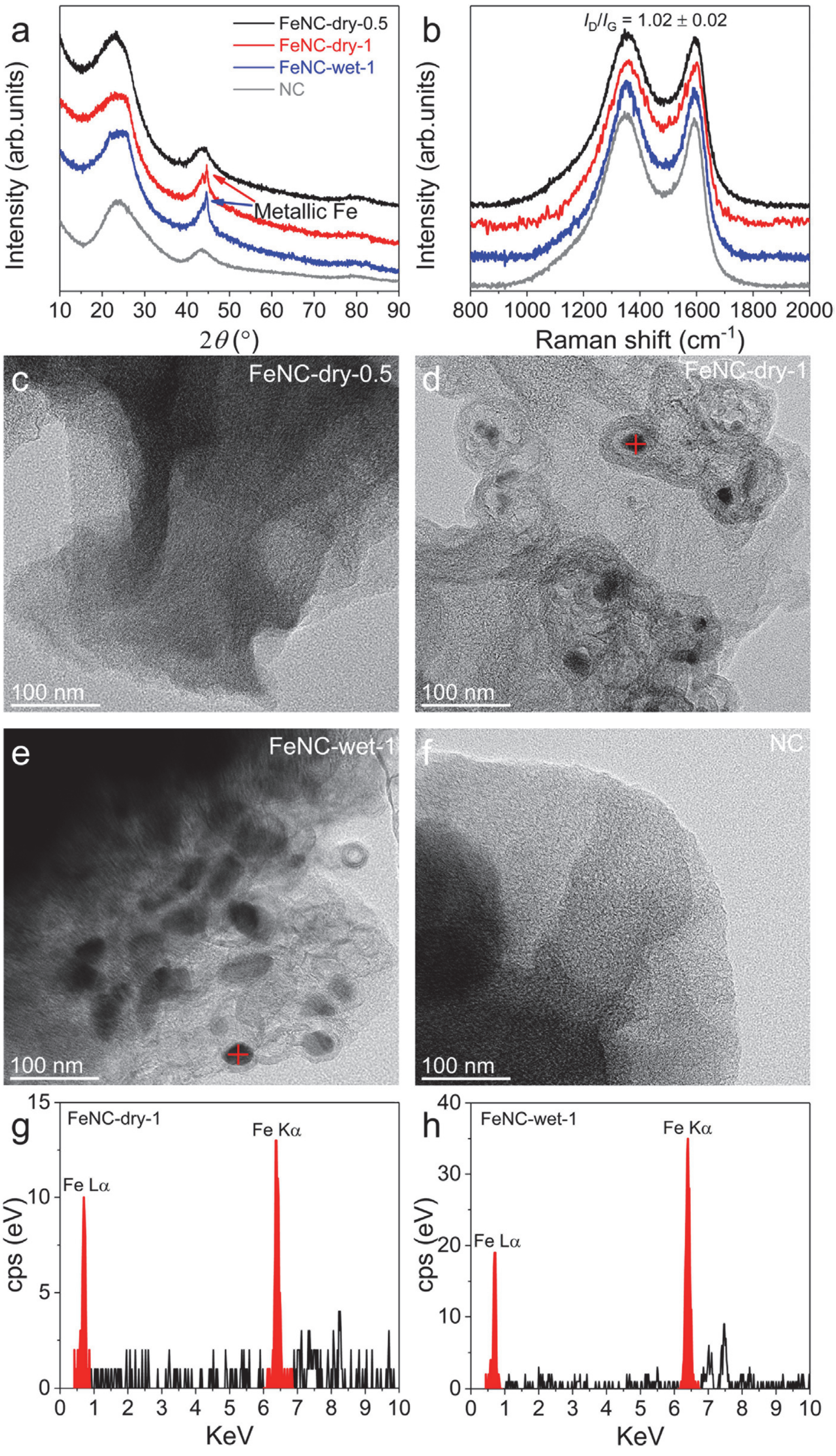

Supplementary Fig. 1. Physical characterisation of the prepared catalysts. **a**, XRD patterns and **b**, Raman spectra of the prepared catalysts. **c**–**f**, TEM images of the prepared catalysts: FeNC-dry-0.5 (**c**), FeNC-dry-1 (**d**), FeNC-wet-1 (**e**), and NC (**f**). Energy-dispersive X-ray

spectroscopy (EDX) results of **g**, FeNC-dry-1 and **h**, FeNC-wet-1. The position, at which the EDX signals were collected, was indicated with a red cross in each TEM image. The XRD results show two broad peaks at ca. 24° and 44° for all the catalysts, which correspond to the (002) and (101) diffraction planes of graphite, respectively. The Raman spectra also reveal the typical D- (@ ~1,356 cm^{-1}) and G-band (@ ~1,588 cm^{-1}) signals, a fingerprint of graphitised carbons with high defect sites (i.e., I_D/I_G = ca. 1.02 ± 0.02). These results indicate successful carbonisation of the precursor mixtures after their pyrolysis at 1,323 K. In the TEM images, TEM-visible metallic particles cannot be found for FeNC-dry-0.5 and NC catalysts due to the absence of Fe species and the predominant presence of atomically-dispersed FeN_xC_y moieties (as confirmed by Mössbauer and EXAFS spectra; Supplementary Figs. 3–4), respectively. On the other hand, the presence of Fe particles (i.e., metallic Fe, Fe oxide, or carbide, as confirmed by Mössbauer spectra; Supplementary Fig. 3) can be found in FeNC-dry-1 and FeNC-wet-1, most of which are protected by carbon layers. However, their crystallographic patterns are hardly detectable in XRD results probably due to a combination of low Fe contents and small particle size. The diffraction peaks that emerged from the bulk Fe phases are detected at ca. 43.6° and 44.7° corresponding to metallic Fe.

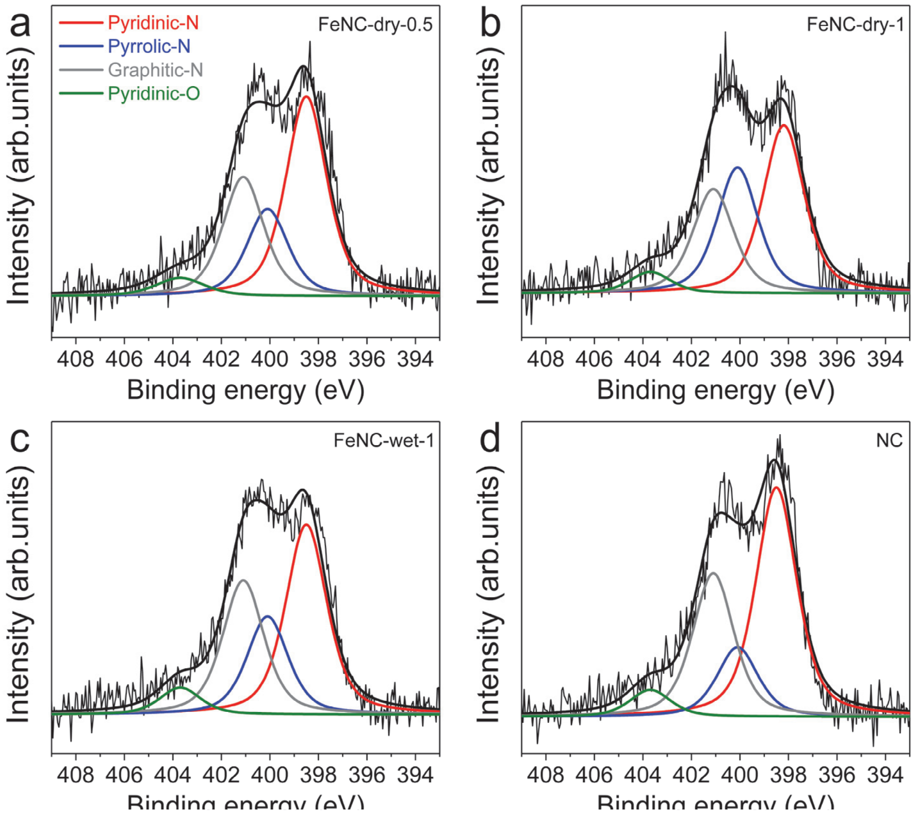

Supplementary Fig. 2. XPS-N$_{1s}$ spectra of the **a**, FeNC-dry-0.5 and **b**–**d**, other control catalysts: FeNC-dry-1 (**b**), FeNC-wet-1 (**c**), and NC (**d**). The peaks are deconvoluted with four N-components: pyridinic-N (red; 398.5 eV), pyrrolic-N (blue; 400.1 eV), graphitic-N (gray; 401.1 eV) and pyridinic-O (green; 403.7 eV).[28]

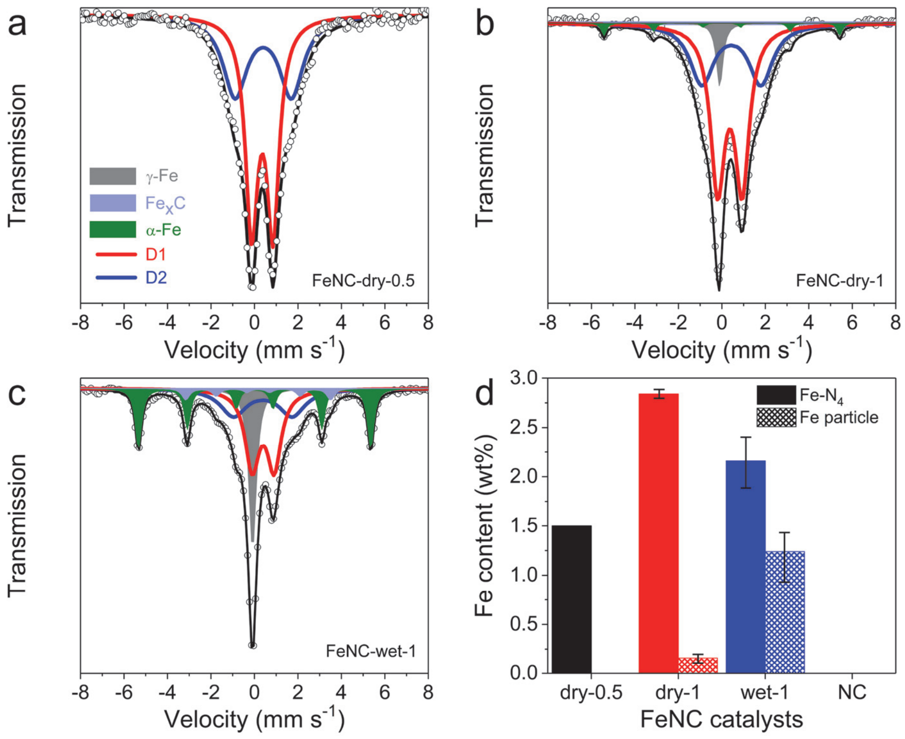

Supplementary Fig. 3. ^{57}Fe Mössbauer absorption spectra and their fit with five spectral components for **a**, FeNC-dry-0.5, **b**, FeNC-dry-1, and **c**, FeNC-wet-1. **d**, Absolute Fe contents of the two main Fe sub-groups (i.e., FeN_xC_y and bulk Fe particles) in the catalysts, which were estimated from the Mössbauer spectrum fittings (Supplementary Table 1).[29] Error bars indicate the uncertainty from the Mössbauer fitting parameters.

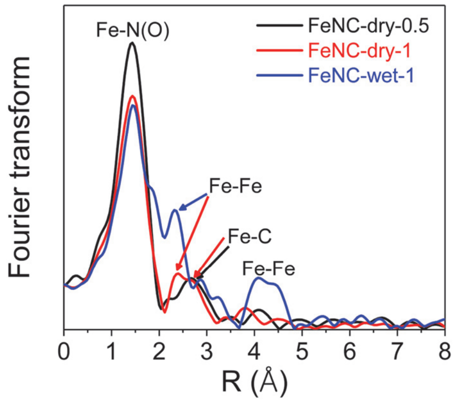

Supplementary Fig. 4. Fourier transformations of the experimental EXAFS spectra of FeNC-dry-0.5 and other control catalysts (FeNC-dry-1 and FeNC-wet-1). The Fourier transformations are not corrected for phase shift.

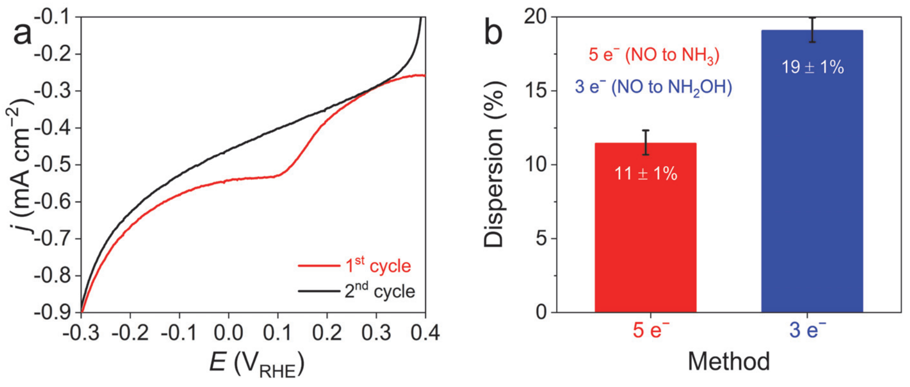

Supplementary Fig. 5. Determination of catalyst site density of FeNC-dry-0.5. **a**, Reductive stripping voltammograms of nitrosyl-poisoned FeNC-dry-0.5. **b**, Fe dispersion, i.e., Fe content exposed to the catalyst surface, estimated from the stripping results. It is assumed that the reductive stripping of nitrosyl ligand leads to a formation of either ammonia (five-electron transfer) or hydroxylamine (three-electron transfer). For the quantification of active site density (i.e., FeN_xC_y moieties) of FeNC-dry-0.5, an in situ electrochemical method, first developed by Kucernak group,[30] was employed. This method is based on the reductive stripping of nitrosyl ligand on FeN_xC_y moieties, the charge of which is directly converted to the number of active sites. For this calculation, the number of electrons transferred during the nitrosyl ligand reduction is one of the key variables, and in the Kucernak group's report its reduction to NH_3 (i.e., five-electron transfer) was assumed. However, in the present work, we have shown that FeNC-dry-0.5 predominantly catalyses NO (or nitrosyl ligand) reduction to NH_2OH even if experimental conditions between the present and Kucernak group's works are not fully identical (Note: in the present work, the quantification analysis was performed at a condition reported by Kucernak group, but NORR electrocatalysis was done under more acidic conditions). Therefore, we cannot distinguish between selective NH_2OH or a competitive NH_2OH/NH_3 production in the present case. On the other hand, its one-electron reduction to N_2O was not considered in the stripping work due to the absence of free NO molecule in the electrolyte, which is required for the N_2O formation.[31] This result thus indicates that Fe dispersion on the catalyst surface is ca. 19% (assuming selective NH_2OH formation) or lower (assuming competitive NH_2OH/NH_3 formation). Error bars indicate the standard error from the three repeated measurements.

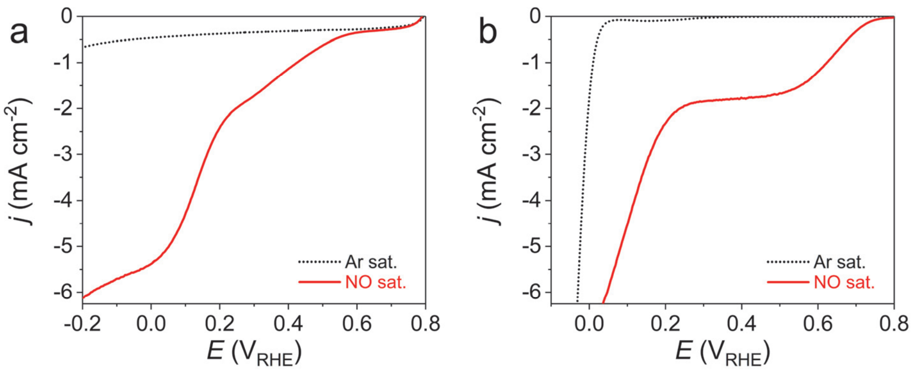

Supplementary Fig. 6. Linear sweep voltammetry (LSV) curves of **a**, FeNC-dry-0.5 and **b**, polycrystalline Pt measured in Ar- and NO-saturated 0.1 M $HClO_4$ electrolytes. The NORR polarisation curves were obtained after subtraction of the currents measured in the Ar-saturated 0.1 M $HClO_4$ electrolyte to remove capacitive currents.

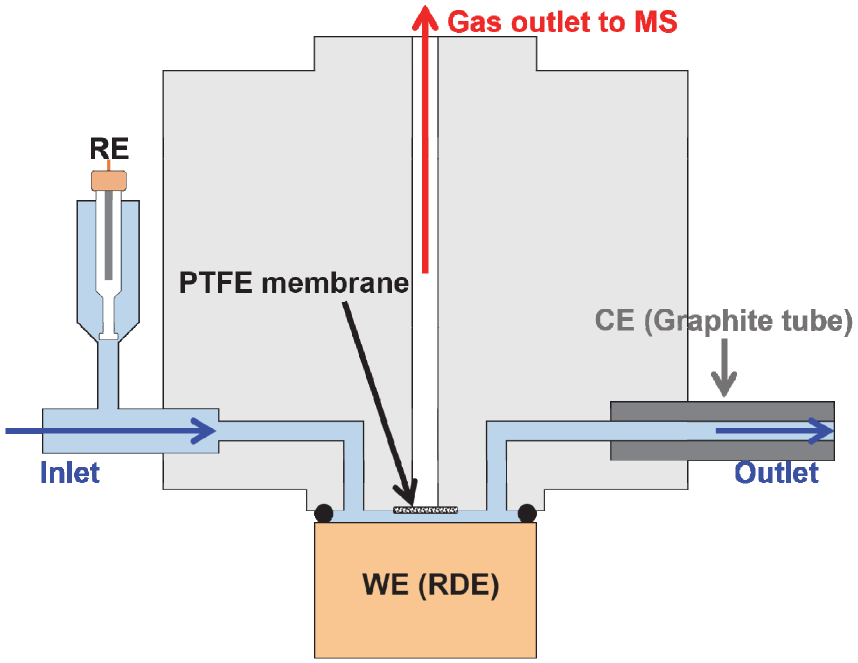

Supplementary Fig. 7. Scheme of the online scanning flow cell (SFC) used for the differential electrochemical mass spectrometry (DEMS) setup. During the NORR, gaseous/volatile products evaporate through a hydrophobic polytetrafluoroethylene (PTFE) membrane, which is positioned ca. 100 μm away from the electrode, traveling into the vacuum system of the mass spectrometer.

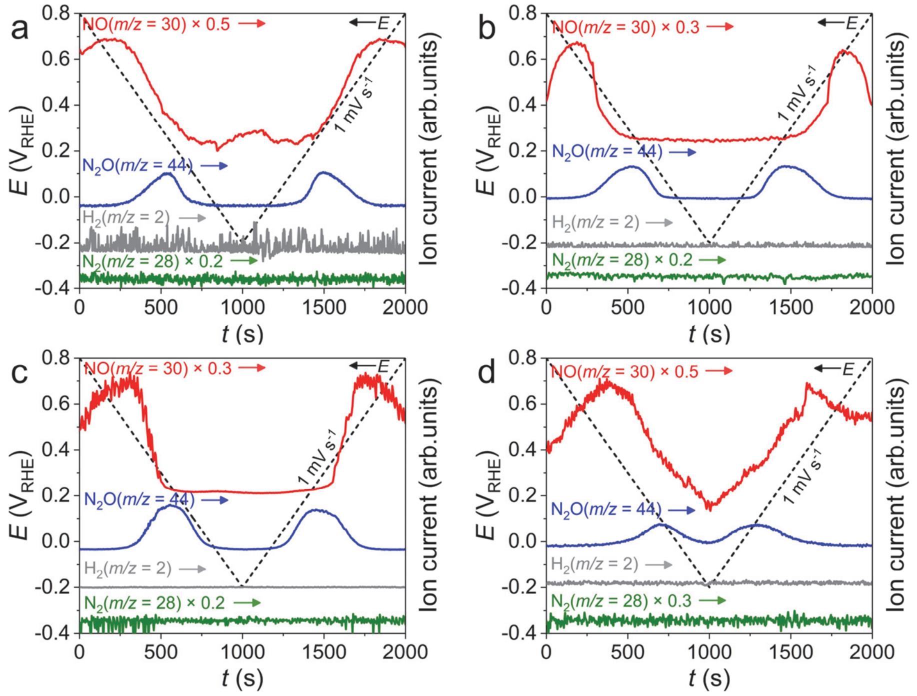

Supplementary Fig. 8. Online SFC/DEMS results measured at a potentiodynamic condition with **a**, FeNC-dry-0.5 and **b**–**d**, other control catalysts: FeNC-dry-1 (**b**), FeNC-wet-1 (**c**), and NC (**d**). Gaseous/volatile NORR products were recorded during a cyclic voltammetry (CV) from 0.8 to −0.2 V_{RHE} with a 1 mV s^{-1} scan rate. Ion currents from NO, N_2O, H_2, and N_2 were monitored at m/z = 30, 44, 2, and 28, respectively.

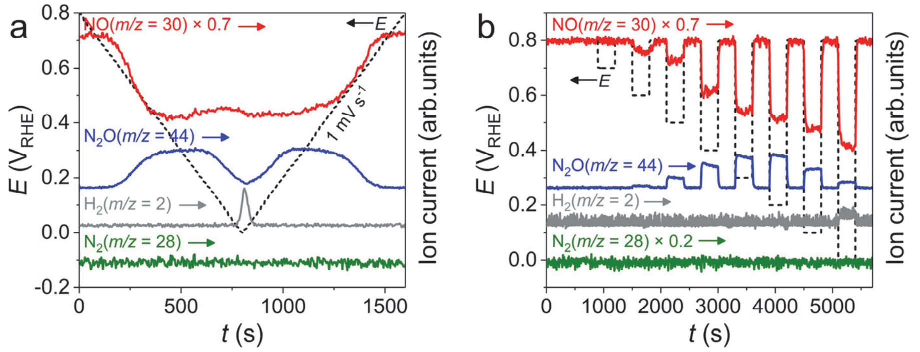

Supplementary Fig. 9. Online SFC/DEMS result of NORR electrocatalysis on polycrystalline Pt. Gaseous/volatile NORR products were recorded during **a**, a CV with a 1 mV s^{-1} scan rate and **b**, a stepwise chronoamperometry (CA) experiment from 0.8 to 0 V_{RHE}. Ion currents from NO, N_2O, H_2, and N_2 were monitored at m/z = 30, 44, 2, and 28, respectively.

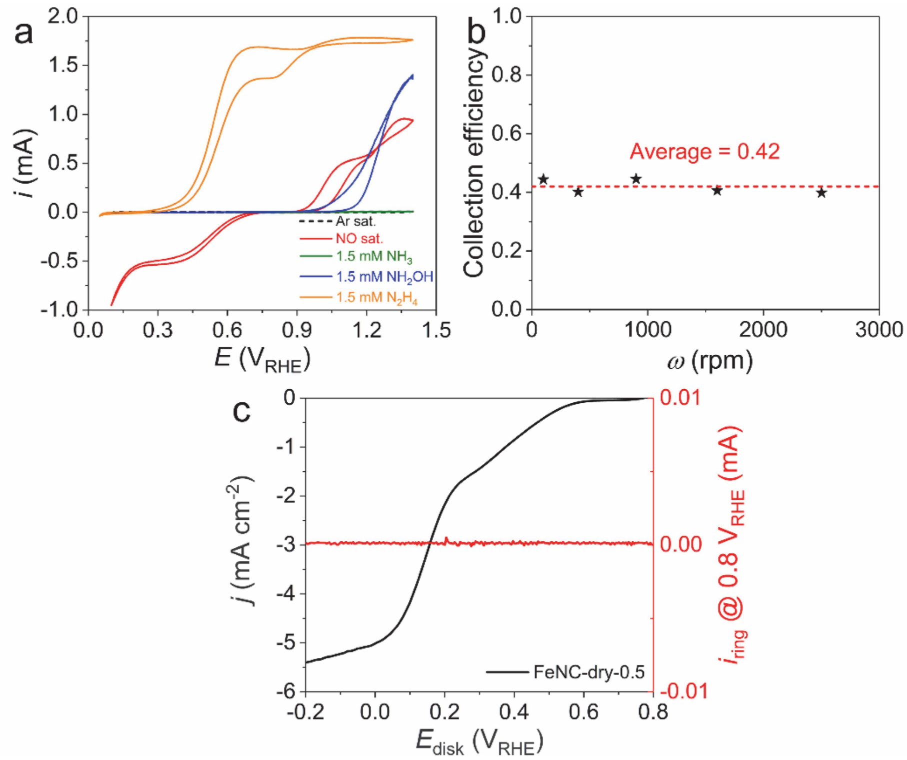

Supplementary Fig. 10. RRDE measurements. **a**, CV results of Pt ring electrode with various electrolyte conditions. Electrolytes are Ar-saturated 0.1 M $HClO_4$ solutions with and without 1.5 mM NH_3/NH_2OH/N_2H_4 and NO-saturated (ca. 1.3 mM) 0.1 M $HClO_4$ solution. The RRDE was rotated at 1,600 rpm. **b**, Collection efficiency of Pt ring in RRDE, which was estimated by a $Fe(CN)_6^{3-}$/$Fe(CN)_6^{4-}$ redox reaction. **c**, RRDE analysis during NORR on FeNC-dry-0.5 catalyst with ring electrode potential at 0.8 V_{RHE}. The black curve indicates the NORR polarisation curve of FeNC-dry-0.5 and red curve indicates the Pt ring current.

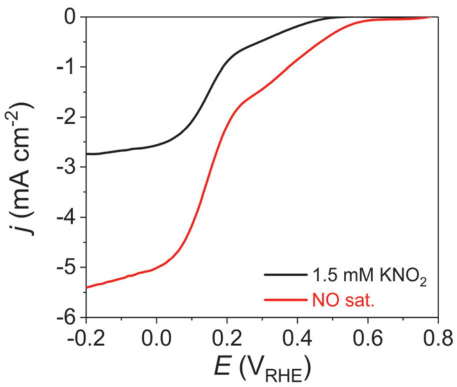

Supplementary Fig. 11. Polarisation curves of FeNC-dry-0.5 in an Ar-saturated 0.1 M $HClO_4$ electrolyte with 1.5 mM KNO_2. That in a NO-saturated 0.1 M $HClO_4$ electrolyte is shown for comparison. Due to the safety issue in the Pohang Accelerator Laboratory beamline, the NO-saturated electrolyte cannot be used for in situ X-ray absorption near edge structure (XANES) analysis. Hence, the 0.1 M $HClO_4$ + 1.5 mM KNO_2 electrolyte was alternatively employed, in which NO can be produced by chemical decomposition of nitrite and dissolved in the electrolyte.[30,32,33] As can be seen in the results, a shape of the polarisation curve obtained in the 0.1 M $HClO_4$ + 1.5 mM KNO_2 electrolyte is almost identical with that in the NO-saturated $HClO_4$ electrolyte, but shows a halved current density probably due to its lower NO concentration than NO-saturated one. The ratio of j_d values at the 1st and 2nd reduction regions is also almost 3. Therefore, all the results imply that this electrolyte can be alternatively used for in situ XANES analysis to understand oxidation states of active Fe centre during the NORR electrocatalysis.

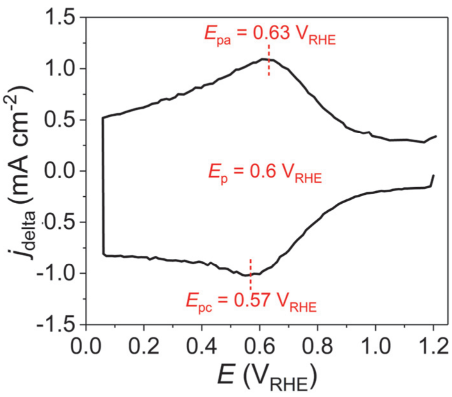

Supplementary Fig. 12. Square-wave voltammogram (SWV) of FeNC-dry-0.5 in an Ar-saturated 0.1 M $HClO_4$ electrolyte. The result shows a clear Fe^{II}/Fe^{III} redox couple at $E_p = 0.6$ V_{RHE} ($E_{pa} = 0.63$ V_{RHE} and $E_{pc} = 0.57$ V_{RHE}). Hence, the result indicates that oxidation state of active FeN_xC_y moieties becomes Fe^{II} at the reductive NORR conditions.

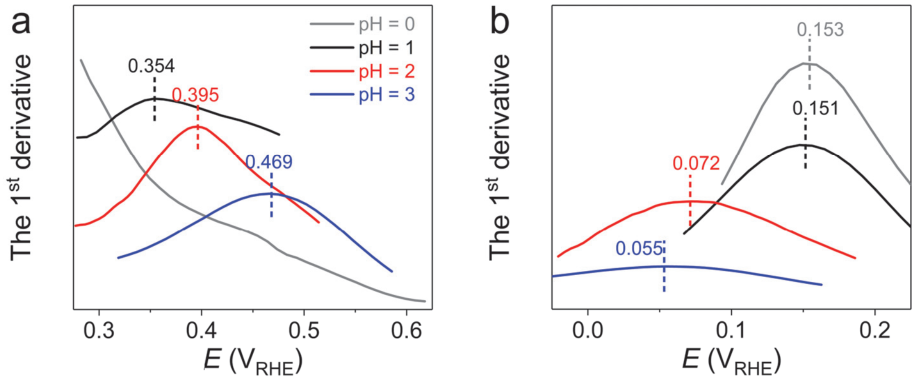

Supplementary Fig. 13. The 1st derivatives of NORR polarisation curves at the **a**, 1st and **b**, 2nd NORR regions. The $E_{1/2}$ of NORR polarisation curves was estimated to a potential at the peak maximum of the 1st derivative.

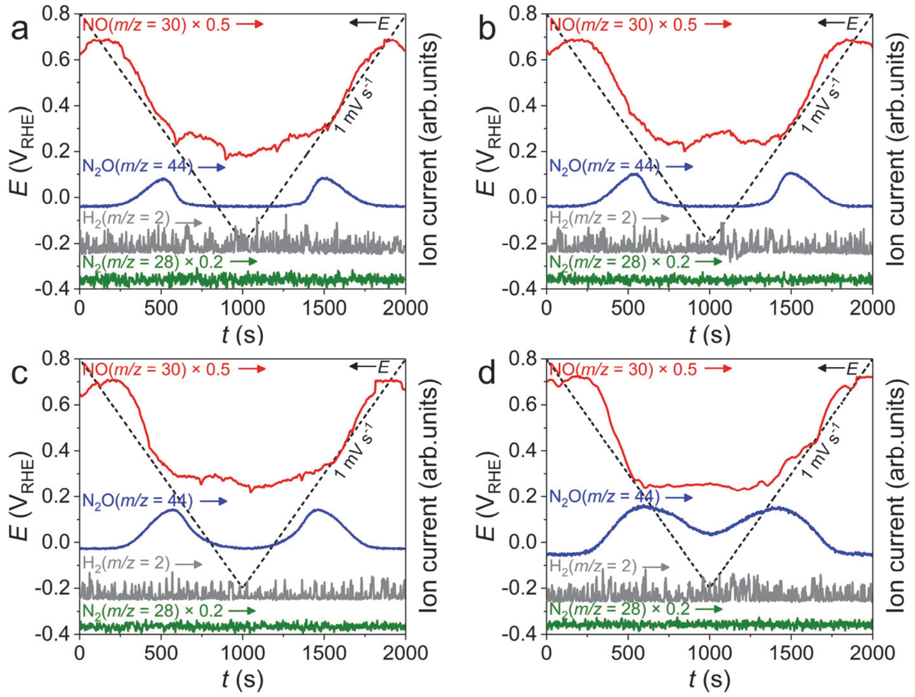

Supplementary Fig. 14. Online SFC/DEMS results of FeNC-dry-0.5 measured at various electrolyte pH values. **a**, NO-saturated 1 M $HClO_4$ (pH 0), **b**, 0.1 M $HClO_4$ (pH 1), **c**, 0.01 M $HClO_4$ + 0.09 M $KClO_4$ (pH 2), and **d**, 0.001 M $HClO_4$ + 0.099 M $KClO_4$ (pH 3) solutions were used as the electrolytes. Gaseous/volatile NORR products were recorded during a CV from 0.8 to −0.2 V_{RHE} with a 1 mV s^{-1} scan rate. Ion currents from NO, N_2O, H_2, and N_2 were monitored at *m*/*z* = 30, 44, 2, and 28, respectively.

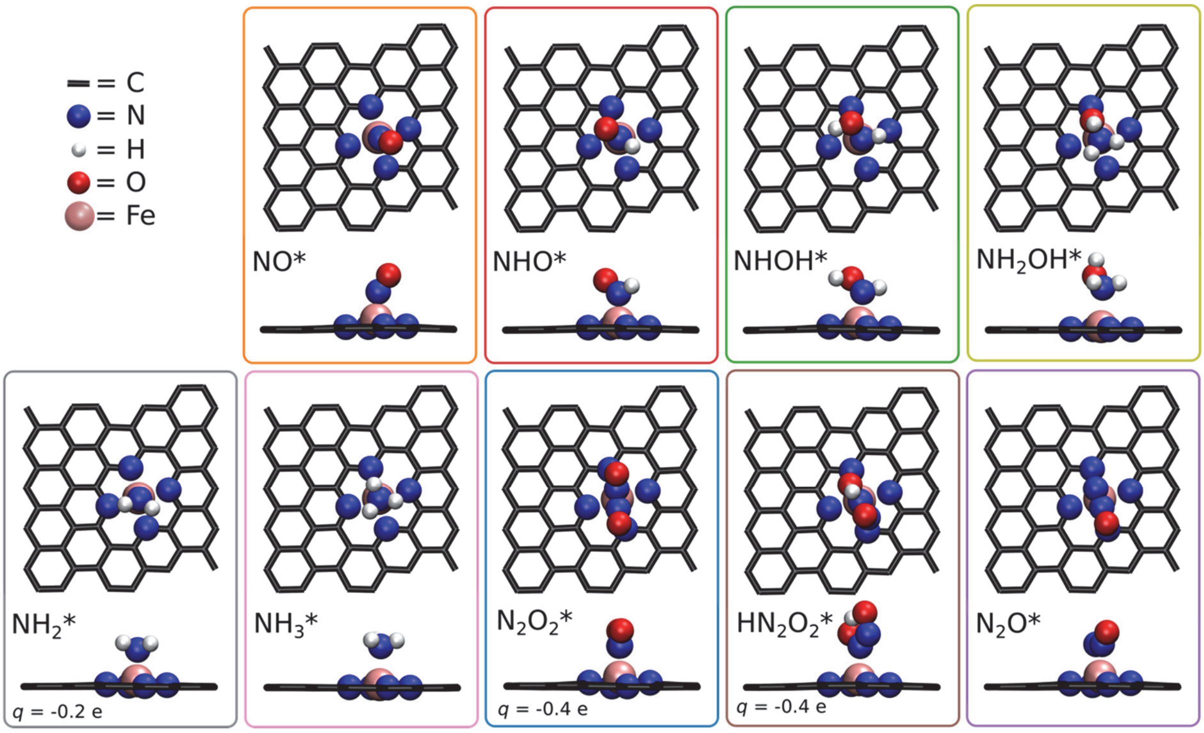

Supplementary Fig. 15. Optimised unit cell structures of all intermediates. The structures were obtained from the zero surface charge calculations, despite some cases, which were not stable at this charge. For those cases, usually a more negative surface charge was required, and the figure shows the structure corresponding to the least negative charge (depicted by the inset) required to bind the adsorbate.

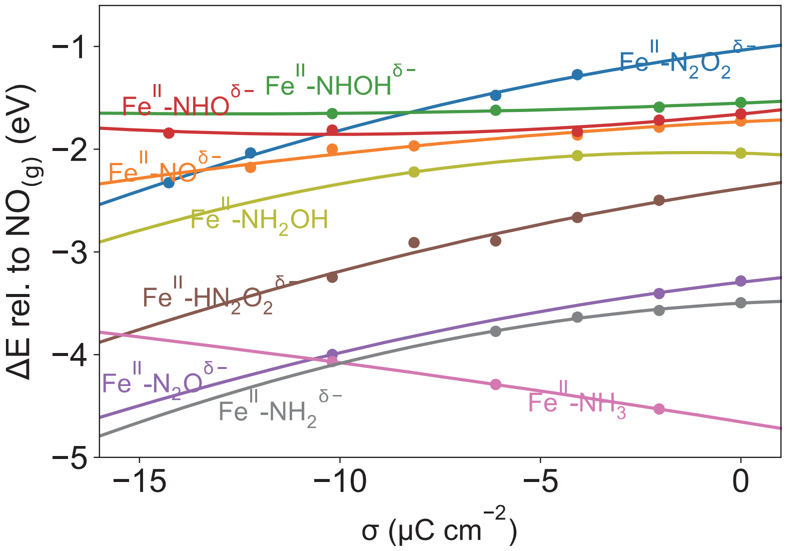

Supplementary Fig. 16. Surface charge dependence of all reaction intermediates based on RPBE calculations with implicit solvent and a planar counter charge. The energies were referenced to $NO_{(g)}$, but without addition of the finite temperature corrections and ZPE.

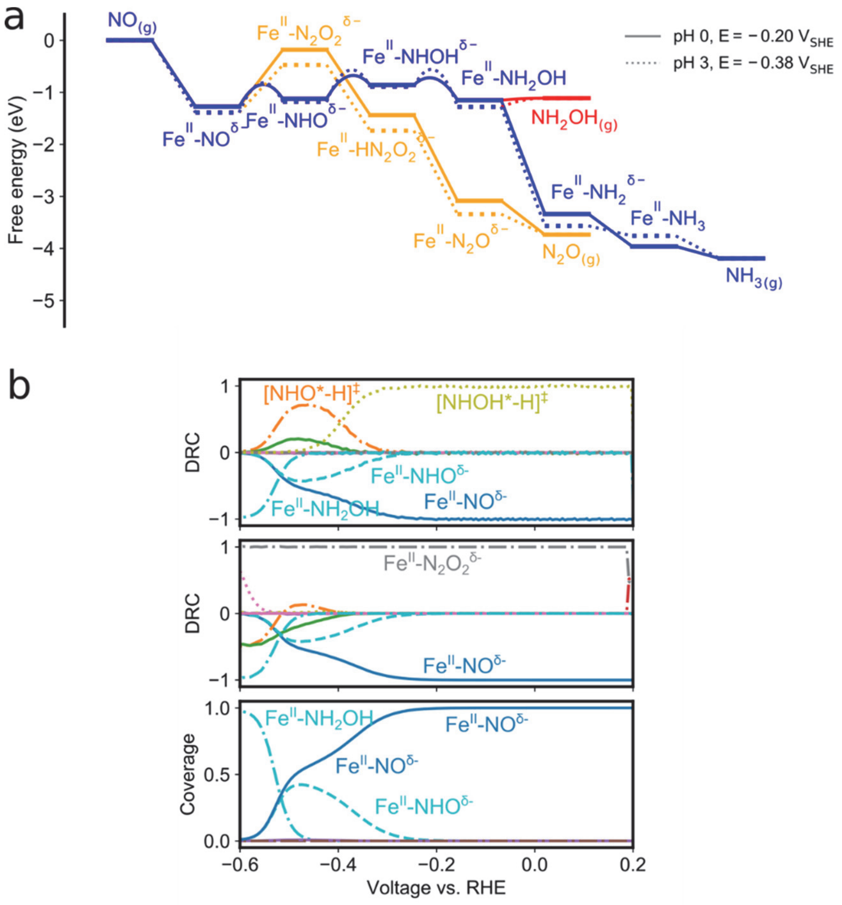

Supplementary Fig. 17. a, Free energy diagram based on RPBE calculations for NORR to the three different products observed experimentally shown at −0.2 V_{RHE} for two different pH values. The x-axis corresponds to the overall reaction coordinate being decomposed into the elementary steps (containing both PCET and chemical steps). The free energy diagram has been evaluated without accounting for pressures and coverages. **b**, Degree of rate-control (DRC) and coverage analysis of the micro-kinetic studies using adsorption energies based on RPBE functional. The upper and central panel shows the DRC for NH_2OH and N_2O formation, respectively. The DRC indicates how strongly an intermediate influences the product formation rate (DRC = +1 (−1) means that stabilisation (destabilisation) of the intermediate enhances the reaction rate). All intermediates have been considered that at least partially in the considered potential range had a DRC>0.1. The lower panel shows all non-zero coverages of intermediates over the whole potential window.

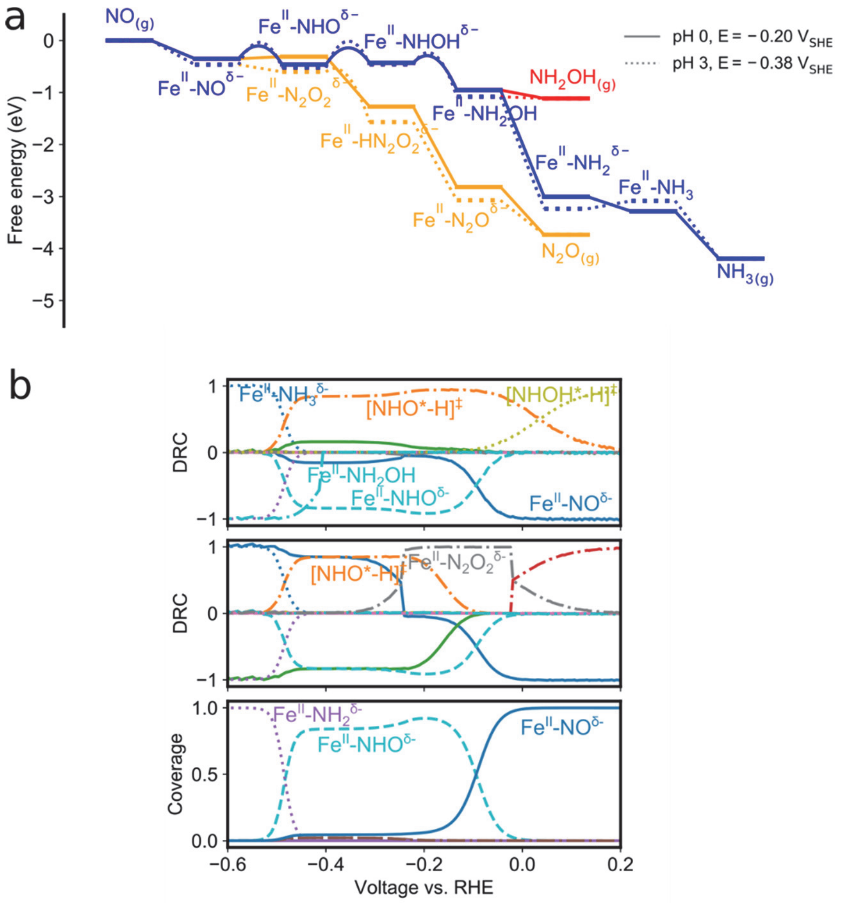

Supplementary Fig. 18. a, Free energy diagram based on RPBE+U2 calculations for NORR to the three different products observed experimentally shown at −0.2 V_{RHE} for two different pH values. The x-axis corresponds to the overall reaction coordinate being decomposed into the elementary steps (containing both PCET and chemical steps). The free energy diagram has been evaluated without accounting for pressures and coverages. **b**, DRC and coverage analysis of the micro-kinetic studies using adsorption energies based on RPBE+U2 functional. The upper and central panel shows the DRC for NH_2OH and N_2O formation, respectively. The DRC indicates how strongly an intermediate influences the product formation rate (DRC = +1 (−1) means that stabilisation (destabilisation) of the intermediate enhances the reaction rate). All intermediates have been considered that at least partially in the considered potential range had a DRC>0.1. The lower panel shows all non-zero coverages of intermediates over the whole potential window.

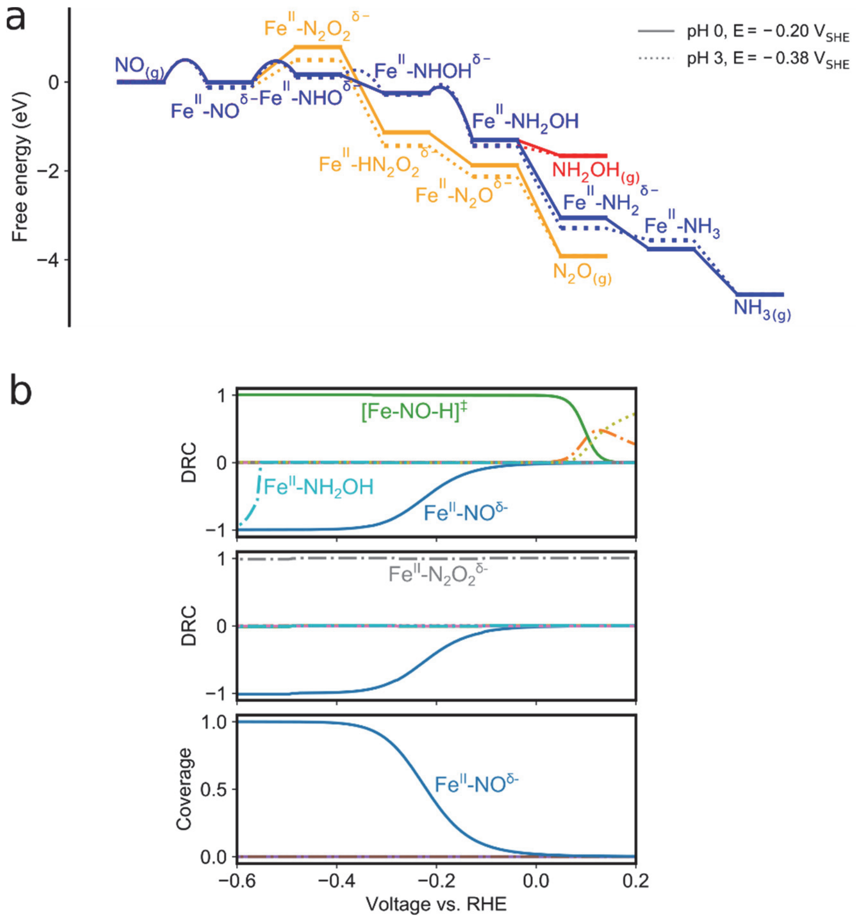

Supplementary Fig. 19. a, Free energy diagram based on HSE06 calculations for NORR to the three different products observed experimentally shown at −0.2 V_{RHE} for two different pH values. The x-axis corresponds to the overall reaction coordinate being decomposed into the elementary steps (containing both PCET and chemical steps). The free energy diagram has been evaluated without accounting for pressures and coverages. **b**, DRC and coverage analysis of the micro-kinetic studies using adsorption energies based on HSE06 functional. The upper and central panel shows the DRC for NH_2OH and N_2O formation, respectively. The DRC indicates how strongly an intermediate influences the product formation rate (DRC = +1 (−1) means that stabilisation (destabilisation) of the intermediate enhances the reaction rate). All intermediates have been considered that at least partially in the considered potential range had a DRC>0.1. The lower panel shows all non-zero coverages of intermediates over the whole potential window.

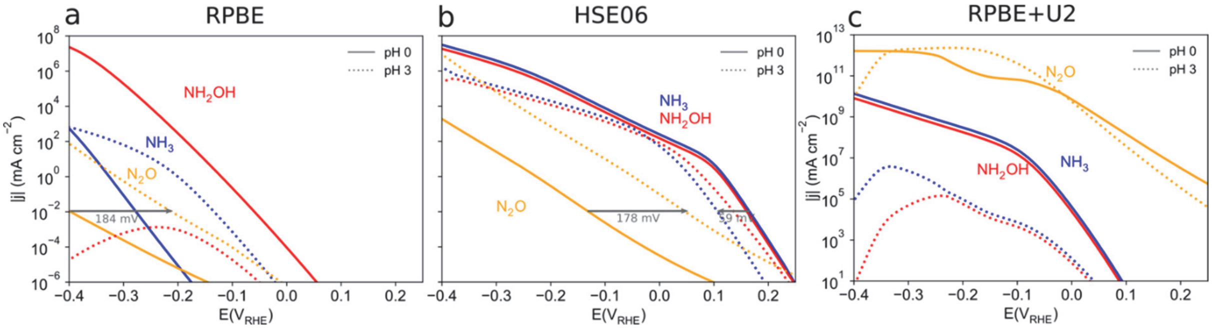

Supplementary Fig. 20. Polarisation curves from micro-kinetic modeling based on adsorption energies calculated with the **a**, RPBE, **b**, HSE06, and **c**, RPBE+U2 functionals. The overpotential shift with pH is indicated by a gray arrow.

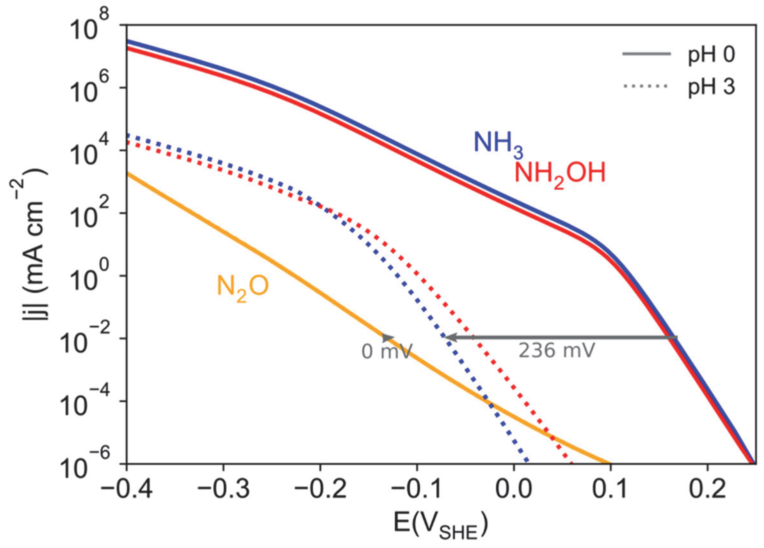

Supplementary Fig. 21. Polarisation curves from micro-kinetic modeling showing the partial current densities of the three products at pH 0 and pH 3 on a SHE scale. Horizontal arrows depict the overpotential shift with pH.

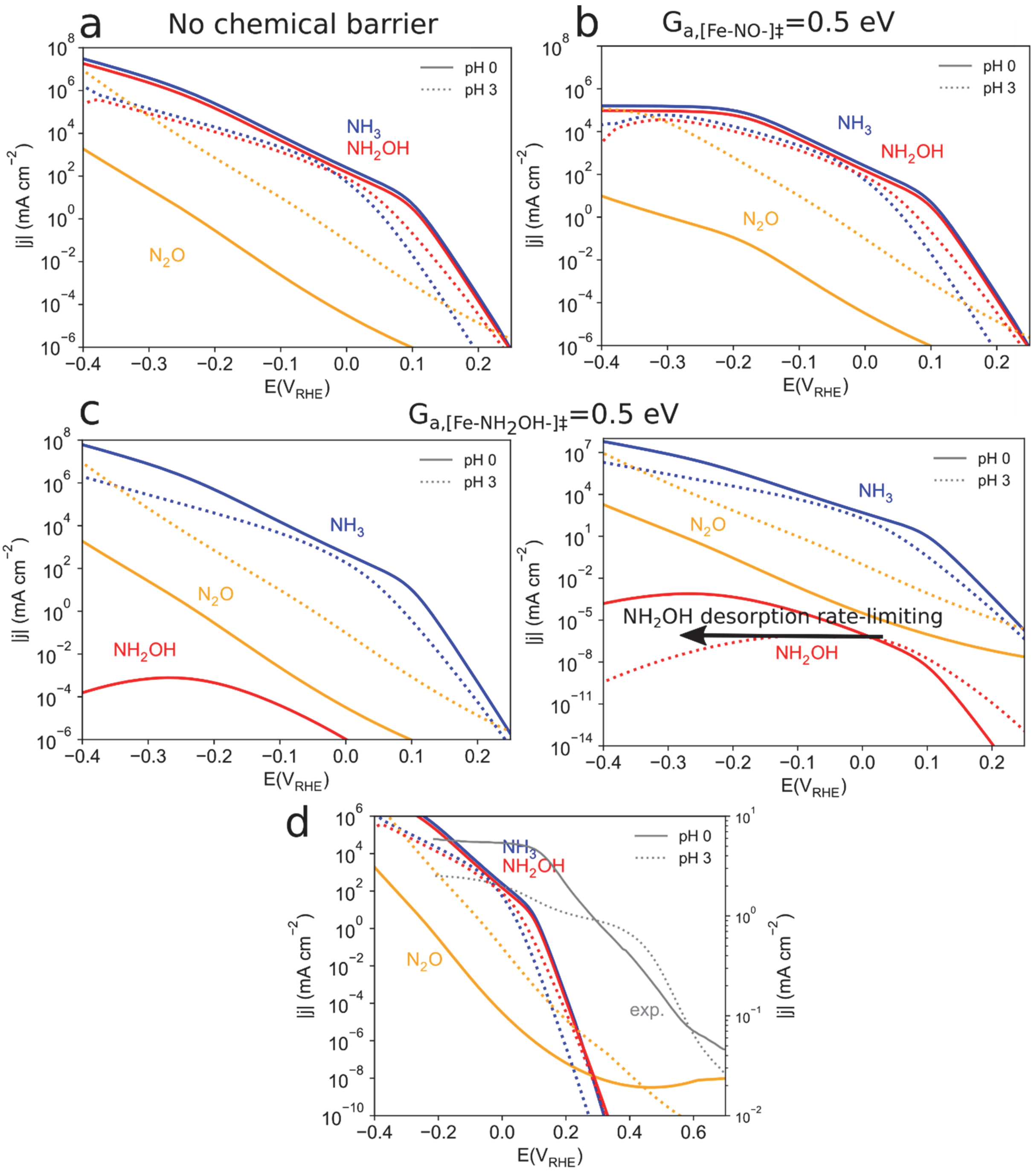

Supplementary Fig. 22. Influence of finite chemical barriers on the polarisation curves obtained from micro-kinetic modeling. **a**, Polarisation curve obtained from the HSE06 without any chemical barriers, **b**, inclusion of NO adsorption barrier of 0.5 eV, and **c**, inclusion of NH_2OH desorption barrier of 0.5 eV. **d**, Comparison of experimental (total current, right y-axis) and theoretical (partial currents, left y-axis) polarisation curves at pH 0 and pH 3.

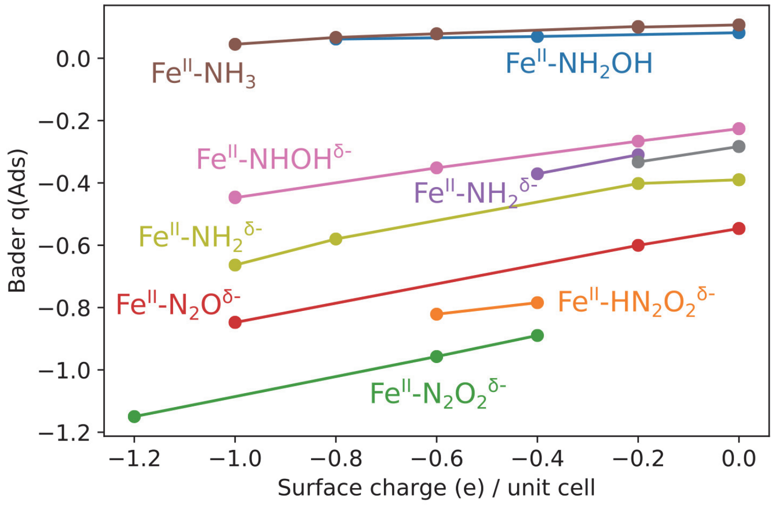

Supplementary Fig. 23. Partial Bader charge of the adsorbates as a function of surface charge/unit area.

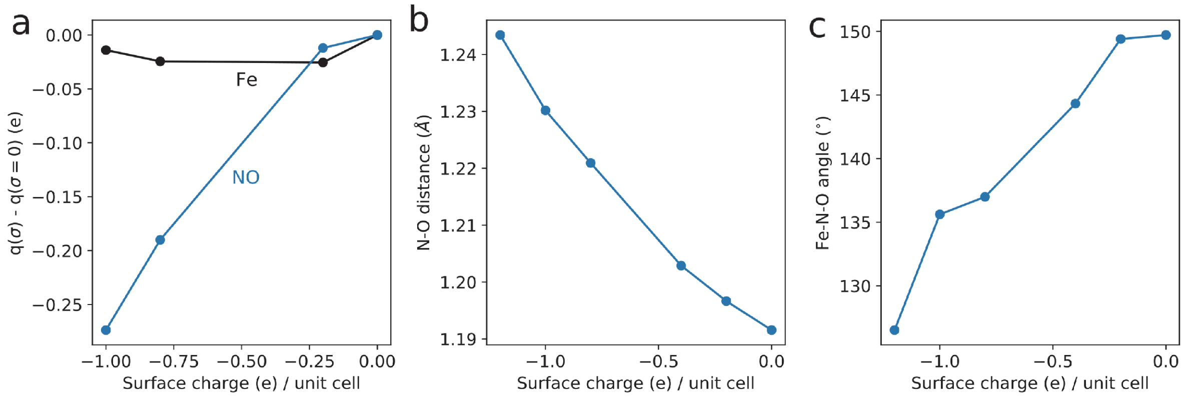

Supplementary Fig. 24. Calculated properties for the Fe^{II} -$NO^{\delta-}$ state. **a**, Change of the Bader charges relative to the PZC of the NO adsorbate and the Fe centre in the optimised NO* state as a function of surface charge per unit cell. **b**, N-O bond distance and **c**, Fe-N-O angle in the optimised NO* state as a function of applied surface charge per unit cell.

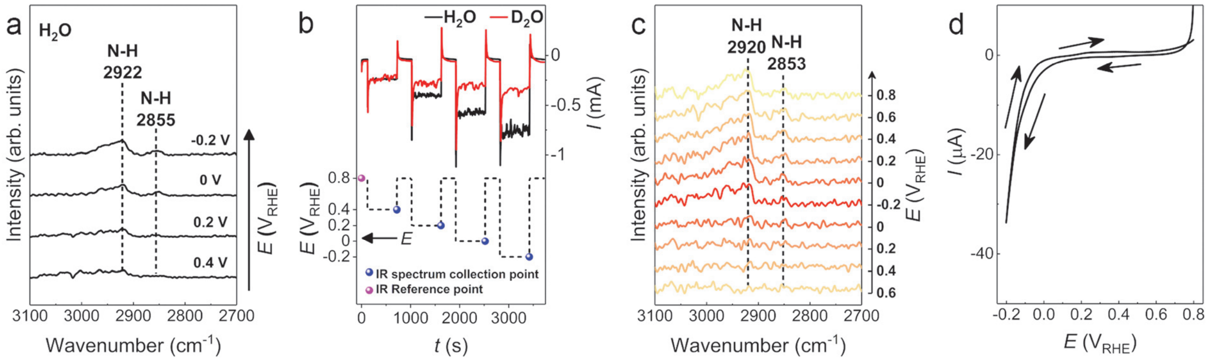

Supplementary Fig. 25. a, In situ ATR-SEIRAS analysis of FeNC-dry-0.5 measured in a NO-saturated 1 mM $HClO_4$ + 0.1 M $KClO_4$/H_2O electrolyte. The IR spectra were collected at constant potentials of 0.4, 0.2, 0, and −0.2 V_{RHE} with a reference spectrum at 0.8 V_{RHE}. **b**, Potential and current profiles during the in situ ATR-SEIRAS analysis for (**a**). **c**, In situ ATR-SEIRAS analysis of Au thin film measured in a 1 mM $HClO_4$ + 0.1 M $KClO_4$ + 1 mM NH_2OH/H_2O electrolyte. The IR spectra were collected at CV from 0.8 to −0.2 V_{RHE}. **d**, Potential and current profiles during the in situ ATR-SEIRAS analysis for (**c**). The N-H bond stretches in Fe^{II}-NH_2OH were clearly observed at 2,922 and 2,855 cm^{-1} (Supplementary Fig. 25a), which corresponded to those of NH_2OH on Au film under an applied bias (Supplementary Fig. 25c).[34]

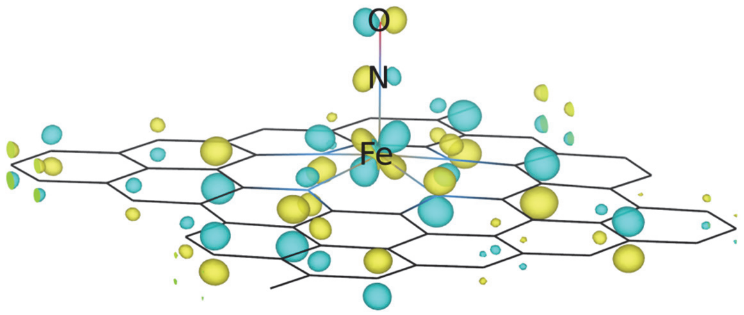

Supplementary Fig. 26. Isosurface of the **s**econd highest occupied molecular orbital (2nd HOMO) of the linear Fe^{II}-$NO^{\delta-}$ state. The contour plot is given for the spin-up MO at the Γ-point and an isovalue of ±0.004 (+ depicted by green, − depicted by blue).

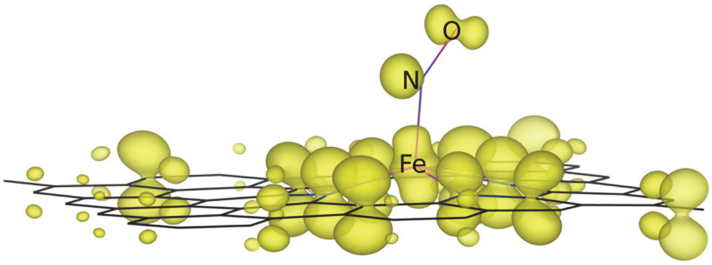

Supplementary Fig. 27. Isosurface of the band projected charge density (HOMO) of the Fe^{II}-$NO^{\delta-}$ state evaluated from the RPBE relaxed geometry using the HSE06 functional. The contour plot is given for the spin-up MO at the Γ-point at a wave function value of ±0.01.

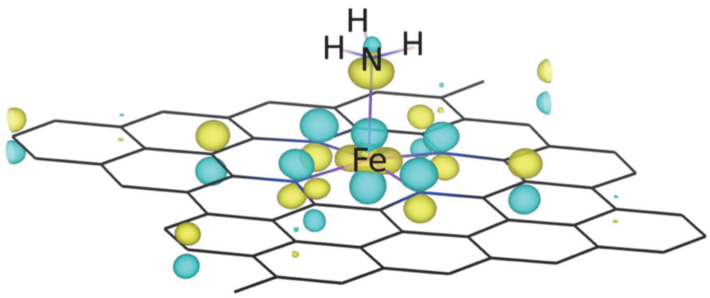

Supplementary Fig. 28. Isosurface of the singly occupied molecular orbital (SOMO) of the Fe^{II}-NH_3 state. The contour plot is given for the spin-up MO at the Γ-point at a wave function value of ±0.004 (+ depicted by green, − depicted by blue).

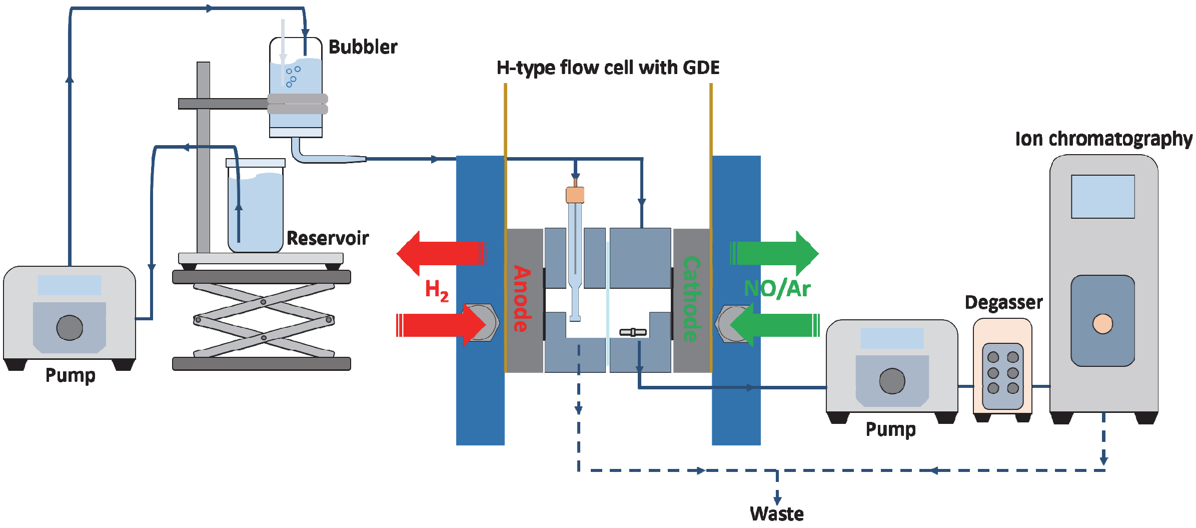

Supplementary Fig. 29. Scheme of the H-type flow cell setup for NH_2OH production in a H_2-NO fuel cell. An Ar-saturated electrolyte continuously flowed into anode and cathode compartments, which were separated by a Nafion 115 membrane. PTFE-treated gas diffusion electrodes (GDEs), on which Pt nanoparticles and FeNC-dry-0.5 were deposited, were used as anode and cathode electrodes, respectively. H_2 and 10% NO/Ar gases were flowed into anode and cathode gas flow channels, where the gases were diffused into the catalyst-electrolyte interfaces through the GDEs. Catholyte outlet was directly connected to ion chromatography (IC), which detected NH_2OH and NH_3 products in their ionised forms (i.e., NH_3OH^+ and NH_4^+, respectively). In the anode compartment, an Ag/AgCl reference electrode was introduced to monitor anode and cathode potentials.

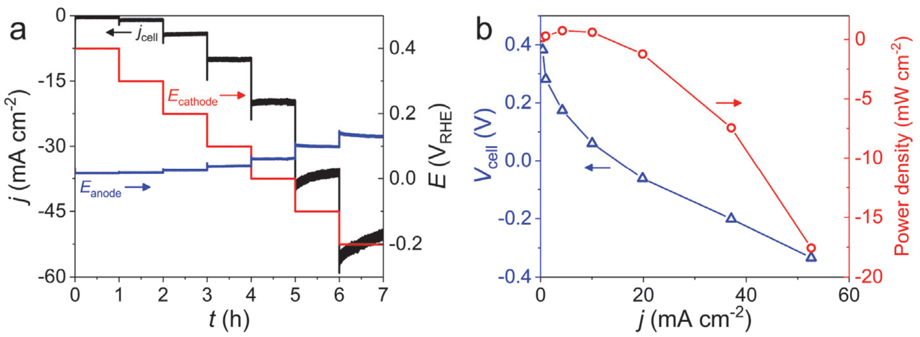

Supplementary Fig. 30. The flow-type H_2-NO fuel cell performance measured with a 1 M $HClO_4$ electrolyte. **a**, Current density and potential profiles during the fuel cell operations. **b**, Fuel cell polarisation curve and calculated power density.

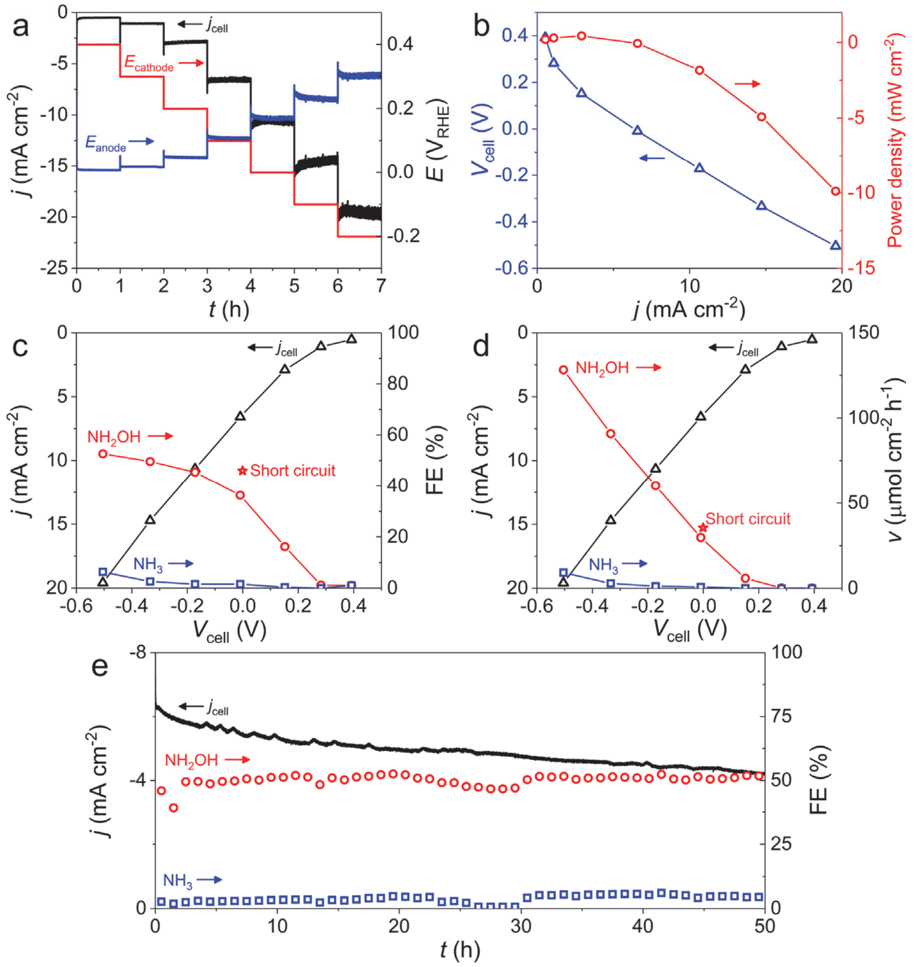

Supplementary Fig. 31. The flow-type H_2-NO fuel cell performance measured with a 0.1 M $HClO_4$ electrolyte. **a**, Current density and potential profiles during the fuel cell operations. **b**, Fuel cell polarisation curve and calculated power density. **c**, Faraday efficiency (FE) and **d**, production rate (*v*) of NH_2OH and NH_3 at various cell voltages. **e**, Current density and FEs during a 50 h long-term durability test at a short-circuit condition.

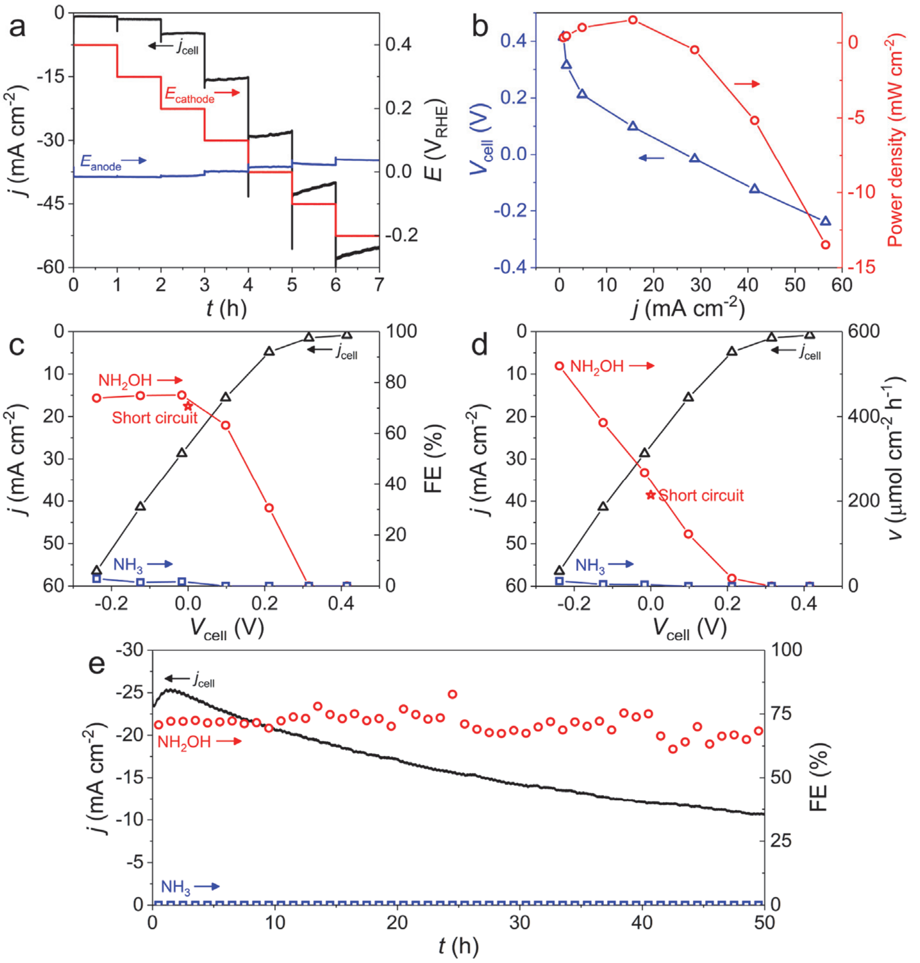

Supplementary Fig. 32. The flow-type H_2-NO fuel cell performance measured with a 3 M $HClO_4$ electrolyte. **a**, Current density and potential profiles during the fuel cell operations. **b**, Fuel cell polarisation curve and calculated power density. **c**, FE and **d**, production rate (v) of NH_2OH and NH_3 at various cell voltages. **e**, Current density and FEs during a 50 h long-term durability test at a short-circuit condition. The non-negligible N_2O formation on GDE, not shown on rotating disk electrode (RDE) at <0.1 V_{RHE} in pH 1 or lower (Fig. 2f), can be interpreted either by the much higher NO activity (i.e., Fe^{II}-HNO + NO + H^+ → Fe^{III} + N_2O + H_2O),[35,36] or by the increased local pH at the catalyst-electrolyte interface.[37-42]

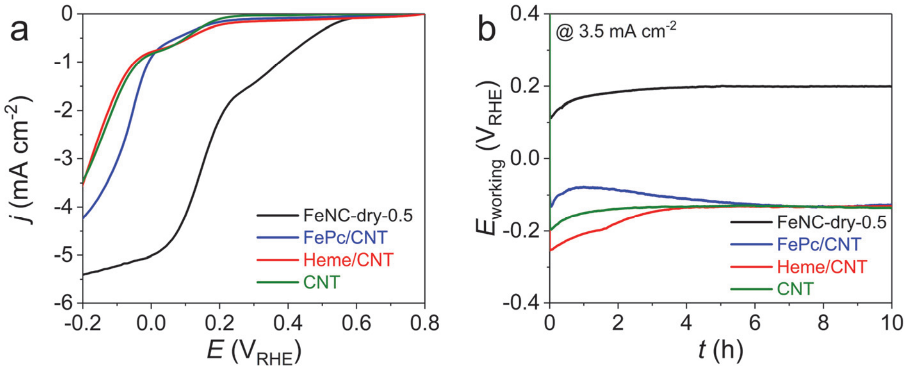

Supplementary Fig. 33. Comparison of activity and stability of FeNC-dry-0.5 and other Fe-based organometallic complexes grafted on carbon nanotube (CNT) support. **a**, NORR polarisation curves and **b**, chronopotentiometry ($j = -3.5$ mA cm^{-2}) results for the catalysts. NORR polarisation curves of Fe-based organometallic complexes (i.e., FePc and heme) grafted on CNT support were collected in a NO-saturated 0.1 M $HClO_4$ electrolyte, and their NORR activity was compared with that of FeNC-dry-0.5 and pristine CNT. In spite of their promising NORR activity reported in literature (at pH 2–12),[2,35,43-45] in highly acidic condition (i.e., pH 1) the organometallic complexes require much higher overpotentials for NORR than that of FeNC-dry-0.5. In particular, NORR activity of heme is almost similar with that of CNT supporting material in the LSV polarisation, even though this catalyst showed highly selective NO-to-NH_2OH conversion at pH 7.[2] Besides the activity, a distinct difference between the organometallic complexes and FeNC-dry-0.5 can be found in their stability, measured at a constant current density of 3.5 mA cm^{-2} for 10 h with the RDE setup. For the FeNC-dry-0.5, the chronopotentiometry result shows very stable NORR at ca. 0.2 V_{RHE} after its stabilisation of ca. 1 h. Similar trend can also be found for CNT. However, the organometallic complexes show unstable NORR activity for the initial 4–6 h operations, which finally converge to the activity of CNT. This result indicates that the organometallic complexes are continuously degraded (e.g., detachment, Fe demetalation, etc.) on CNT. Hence, it can be concluded that FeNC-dry-0.5, on which active FeN_xC_y moieties are covalently bonded with graphite substrate, has good NORR activity and stability in such highly acidic environments (much better than those of organometallic complexes), which are prerequisites for successful NH_2OH production in H_2-NO fuel cells.

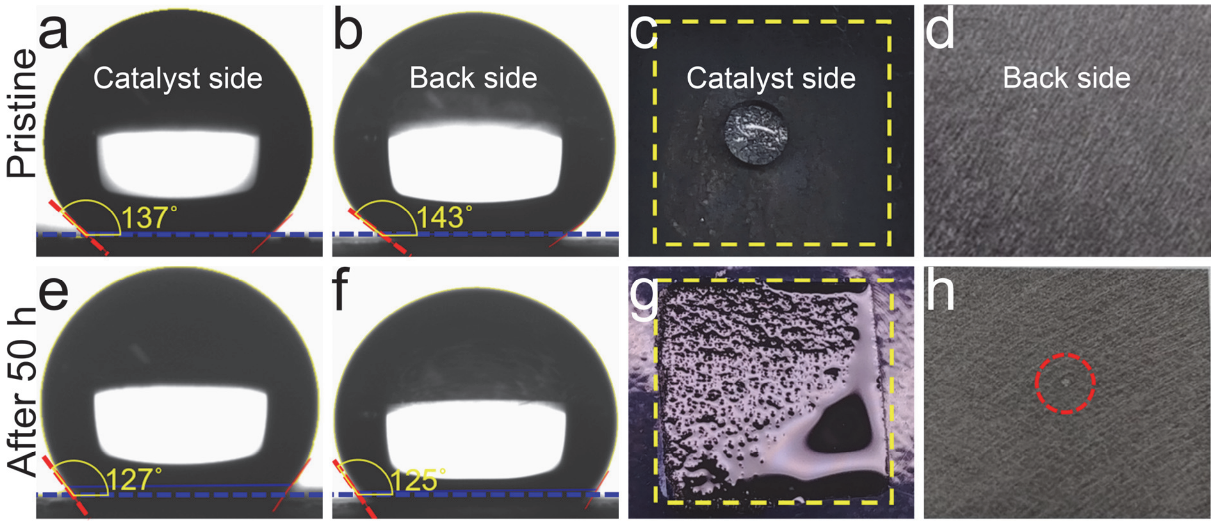

Supplementary Fig. 34. GDE wetting **a–d**, before and **e–h**, after the 50 h durability test of H_2-NO fuel cell with a 1 M $HClO_4$ electrolyte. Representative images of water droplets on the cathode GDE for contact angle measurements: catalyst side (**a**,**e**) and back side (**b**,**f**). Photographs of GDE before and after the durability test: catalyst side (**c**,**g**) and back side (**d**,**h**). The catalyst side faced to the electrolyte and back side faced to gas-flow channel/current collector. Yellow rectangles indicate the active area of catalyst side, un-covered by silicon gasket. A red circle indicates the electrolyte leakage at the back side of GDE after the durability test.

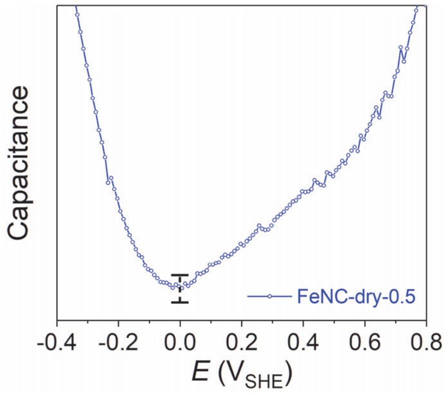

Supplementary Fig. 35. PZC measurement. The capacitance was measured in 10 mM NaF solution with staircase potentiostatic electrochemical impedance spectroscopy (SPEIS) technique. The minimum point of the capacitance (point with dashed bar) indicates the PZC of the FeNC-dry-0.5 (ca. 0 V_{SHE}).

SUPPLEMENTARY TABLES

Supplementary Table 1. Mössbauer fitting parameters and relative contents of five Fe components as estimated from their relative absorption areas of the spectra for FeNC-dry-0.5 and other control catalysts (FeNC-dry-1 and FeNC-wet-1).

Catalysts	Comp.[a]	IS[b] (mm s^{-1})	QS[c] (mm s^{-1})	LW[d] (mm s^{-1})	HF[e] (mm s^{-1})	RA[f] (%)	RC[g] (%)
FeNC-dry-0.5	D1	0.36	0.98	0.69	-	58	61
	D2	0.40	2.59	1.32	-	42	39
FeNC-dry-1	D1	0.38	1.10	0.78	-	58	62.3
	D2	0.43	2.65	1.45	-	34	32.3
	Sextet 1	−0.02	-	0.35	33.6	3	2.2
	Singlet	−0.10	-	0.42	-	5	3.2
FeNC-wet-1	D1	0.40	1.01	0.78	-	36	44
	D2	0.38	2.07	1.45	-	18	20
	Sextet 1	0.01	-	0.35	33.2	23	19
	Sextet 2	0.17	-	0.42	20.7	6	4
	Singlet	−0.08	-	0.42	-	18	13

[a] Components: $Fe^{II}N_4$/C-LS (D1), $Fe^{II}N_4$/C-MS (D2), *α*-Fe (Sextet 1), Fe_xC (Sextet 2), and *γ*-Fe (Singlet).

[b] Isomer shift

[c] Quadrupole splitting

[d] Line width

[e] Hyperfine field

[f] Relative absorption area

[g] Relative Fe contents, calculated from the relative area of Fe components in the Mössbauer spectra with the Lamb-Mössbauer factors of each component.[29]

Supplementary Table 2. Adsorption energies of all intermediate states using the RPBE, RPBE+U2, and HSE06 functionals as obtained from the RPBE relaxed geometries at zero surface charge and integrated difference of spin up minus spin down electron density (total magnetization) at the charges at which the optimized geometries in Supplementary Fig. 15 were obtained. The RPBE calculation was carried out with implicit solvation, using $\varepsilon_b = 6$, while all other calculations were carried out under a vacuum environment. The energies were referenced to $NO_{(g)}$, but without addition of the finite temperature corrections and ZPE.

Adsorption state	ΔE (RPBE, eV)	ΔE (RPBE+U2, eV)	ΔE (HSE06, eV)	Integrated spin-up – spin-down electron density/total magnetization (Bohr magneton/cell)
Fe^{II}-$NO^{\delta-}$	−1.73	−0.81	−0.46	0.84
Fe^{II}-$NHO^{\delta-}$	−1.66	−1.00	−0.37	0.00
Fe^{II}-$NHOH^{\delta-}$	−1.55	−1.12	−0.94	0.99
Fe^{II}-NH_2OH	−2.04	−1.85	−2.20	1.92
Fe^{II}-$N_2O_2^{\delta-}$	−1.03	−1.17	−0.07	0.06
Fe^{II}-$HN_2O_2^{\delta-}$	−1.77	−1.61	−1.47	2.73
Fe^{II}-$N_2O^{\delta-}$	−3.38	−3.11	−2.17	1.48
Fe^{II}-$NH_2^{\delta-}$	−3.59	−3.26	−3.31	1.02
Fe^{II}-NH_3	−4.75	−4.07	−4.54	2.01

Supplementary Table 3. Operating conditions of fuel cells or electrolyser and NH_2OH production performance.[a]

Electrodes			Operating conditions					NH_2OH production performance			Ref.
Catalyst	Loading (mg cm^{-2})	Area (cm^2)	Electrolyte	E_{cell} (V)	j[b] (mA cm^{-2})	P_{NO}[c] (%)	Flow rate (sccm)	Production rate (μmol cm^{-2} h^{-1})	FE (%)	Stability test (h)	
FeNC-dry-0.5	0.70	1	0.1 M $HClO_4$	0	6.8	10	60	35	45	50	This study
				−0.50	19.6			128	53	-	
			1 M $HClO_4$	0	14.8			105	61	50	
				−0.33	52.6			423	65	-	
			3 M $HClO_4$	0	24.4			215	71	50	
				−0.24	56.5			519	74	-	
-/Gr	0.00	3.1	5 M H_2SO_4	0	0.4	9	10	>1	22	-	[46][d,e]
$FeSO_4$/Gr	0.18				0.8			>1	13		
FeTPP/Gr	0.81				0.3			<1	20		
CoPc/Gr	0.69				0.3			<1	27		
CuPc/Gr	0.70				0.1			0	0		
FePc/Gr	0.69				3.8			41	88		
			5 M H_3PO_4		3.6			32	73		
			5 M $HClO_4$		6.3			60	78		
			2 M KOH		1.4			0	0		
FePc/AC			5 M H_2SO_4		9.2			107	92		
FePc/CW			5 M H_2SO_4		10.9			116	85		
FePc/CB			5 M H_2SO_4		1.7			14	67		
Fe-PANI-AC-1	-	10	3 M H_2SO_4	0	11.5	6	10	46	34	-	[47][d]
				0.01	-			-	-	11	
Fe-PANI-AC-2				0	9.8			27	29	-	
				0.01	-			-	-	11	
FePc/AC				0	7.6			60	80	-	
Fe-PANI-AC-2				0	15.3	18		123	63		
				0.05	13.9			92	53		
				0.1	11.2			63	46		
FePc/AC				0	11.7			44	30		
				0.05	7.4			34	42		
				0.1	6.4			32	40		
AC	-	10	3 M H_2SO_4	0	5.6	6	10	7	22	-	[48][d]
$FePc_{(3.0)}$/AC					12.3			77	55		
$FePc_{(0.3)}$/AC					7.6			60	80		

$FeSalen_{(0.3)}$/AC					5.2			38	51		
AC-pyr					5.8			10	26		
$FePc_{(0.3)}$/AC-pyr					5.6			27	39		
$FeSalen_{(0.3)}$/AC-pyr					6.2			37	54		
AC					9.1	18		21	18		
$FePc_{(0.3)}$/AC					11.7			44	30		
				0.05	7.4			34	42	23.6 h	
				0.1	6.4			32	40	-	
$FeSalen_{(0.3)}$/AC				0	10.6			53	38		
				0.05	8.6			34	43	23.6 h	
				0.1	5.6			0	0	-	
AC-pyr				0	6			19	24		
$FePc_{(0.3)}$/AC-pyr				0	9			21	18		
				0.05	7.1			13	15	23.6 h	
				0.1	4.9			5	8.2	-	
$FeSalen_{(0.3)}$/AC-pyr				0	8.7			21	19		
				0.05	7.7			20	20	23.6 h	
				0.1	5.9			6	8	-	
WC	-	-	0.5 M H_2SO_4	-	21.3	100	221	153	58	-	[49][f]
			1 M H_2SO_4					166	63		
			2 M H_2SO_4					195	74		

[a] All the device operations were performed at room temperature.

[b] Current density

[c] Partial pressure of NO

[d] H_2-NO fuel cells

[e] Catalyst loading, j, NH_2OH production rate, and FE_{NH2OH} were estimated as follows (Note that only metal compounds, not carbon substrate, were defined as catalysts);

$$\text{Catalyst Loading (mg cm}^{-2}) = \frac{\text{Molecular weight of catalyst (g mol}^{-1}) \times \text{Catalyst molar ratio compared to carbon substrate} \times \text{Carbon substrate amount (g)}}{\text{Atomic weight of carbon (12 g mol}^{-1}) \times \text{Geomatric surface area (cm}^2)} \times 1{,}000$$

$$j\ (\text{mA cm}^{-2}) = \frac{\text{Total charge (mC)}}{\text{Geomatric surface area (cm}^2) \times \text{Operating time (s)}}$$

$$\text{NH}_2\text{OH production rate } (\mu\text{mol cm}^{-2}\ \text{h}^{-1}) = \frac{\text{Total NH}_2\text{OH production } (\mu\text{mol})}{\text{Geomatric surface area (cm}^2) \times \text{Operating time (h)}}$$

$$\text{FE}\ (\%) = \frac{3 \times F\ (96{,}485\ \text{C mol}^{-1}) \times \text{Total NH}_2\text{OH production (mol)}}{\text{Total charge (C)}} \times 100$$

[f] H_2O-NO electrolyser, which was operated at a constant current (chronopotentiometry). The NH_2OH production rate was calculated as follow;

$$\text{NH}_2\text{OH production rate}\ (\mu\text{mol cm}^{-2}\ \text{h}^{-1}) = \frac{j\ (\text{mA cm}^{-2}) \times 3{,}600\ (\text{s h}^{-1}) \times \text{FE}\ (\%)}{3 \times F\ (96{,}485\ \text{C mol}^{-1})} \times \frac{1{,}000}{100}$$

SUPPLEMENTARY REFERENCES

1 de Vooys, A. C. A., Koper, M. T. M., van Santen, R. A. & van Veen, J. A. R. Mechanistic study of the nitric oxide reduction on a polycrystalline platinum electrode. *Electrochim. Acta* **46**, 923-930 (2001).

2 de Groot, M. T., Merkx, M., Wonders, A. H. & Koper, M. T. M. Electrochemical reduction of NO by hemin adsorbed at pyrolitic graphite. *J. Am. Chem. Soc.* **127**, 7579-7586 (2005).

3 de Vooys, A. C. A., Beltramo, G. L., van Riet, B., van Veen, J. A. R. & Koper, M. T. M. Mechanisms of electrochemical reduction and oxidation of nitric oxide. *Electrochim. Acta* **49**, 1307-1314 (2004).

4 de Vooys, A. C. A., Koper, M. T. M., van Santen, R. A. & van Veen, J. A. R. Mechanistic study on the electrocatalytic reduction of nitric oxide on transition-metal electrodes. *J. Catal.* **202**, 387-394 (2001).

5 Giannozzi, P. *et al.* QUANTUM ESPRESSO: a modular and open-source software project for quantum simulations of materials. *J. Phys.: Condens. Matter* **21**, 395502 (2009).

6 Hjorth Larsen, A. *et al.* The atomic simulation environment—a Python library for working with atoms. *J. Phys.: Condens. Matter* **29**, 273002 (2017).

7 Hammer, B., Hansen, L. B. & Nørskov, J. K. Improved adsorption energetics within density-functional theory using revised Perdew-Burke-Ernzerhof functionals. *Phys. Rev. B* **59**, 7413-7421 (1999).

8 Monkhorst, H. J. & Pack, J. D. Special points for Brillouin-zone integrations. *Phys. Rev. B* **13**, 5188-5192 (1976).

9 Andreussi, O., Dabo, I. & Marzari, N. Revised self-consistent continuum solvation in electronic-structure calculations. *J. Chem. Phys.* **136**, 064102 (2012).

10 Fumagalli, L. *et al.* Anomalously low dielectric constant of confined water. *Science* **360**, 1339-1342 (2018).

11 Beltramo, G., Giesen, M. & Ibach, H. Anomalous Helmholtz-capacitance on stepped surfaces of silver and gold. *Electrochim. Acta* **54**, 4305-4311 (2009).

12 Noguchi, H., Okada, T. & Uosaki, K. Molecular structure at electrode/electrolyte solution interfaces related to electrocatalysis. *Faraday Discuss.* **140**, 125-137 (2009).

13 Parsons, R. The metal-liquid electrolyte interface. *Solid State Ion.* **94**, 91-98 (1997).

14 Toney, M. F. *et al.* Distribution of water molecules at Ag(111)/electrolyte interface as

studied with surface X-ray scattering. *Surf. Sci.* **335**, 326-332 (1995).

15 Toney, M. F. *et al.* Voltage-dependent ordering of water molecules at an electrode–electrolyte interface. *Nature* **368**, 444-446 (1994).

16 Ringe, S. *et al.* Understanding cation effects in electrochemical CO_2 reduction. *Energy Environ. Sci.* **12**, 3001-3014 (2019).

17 Ringe, S. *et al.* Double layer charging driven carbon dioxide adsorption limits the rate of electrochemical carbon dioxide reduction on gold. *Nat. Commun.* **11**, 33 (2020).

18 Kresse, G. & Furthmüller, J. Efficient iterative schemes for *ab initio* total-energy calculations using a plane-wave basis set. *Phys. Rev. B* **54**, 11169-11186 (1996).

19 Heyd, J., Scuseria, G. E. & Ernzerhof, M. Hybrid functionals based on a screened Coulomb potential. *J. Chem. Phys.* **118**, 8207-8215 (2003).

20 Sudarshan, V. *et al.* Dipole field interactions determine the CO_2 reduction activity of 2D FeNC single atom catalysts. *ACS Catal.* **10**, 7826-7835 (2020).

21 Blöchl, P. E. Projector augmented-wave method. *Phys. Rev. B* **50**, 17953-17979 (1994).

22 Kresse, G. & Joubert, D. From ultrasoft pseudopotentials to the projector augmented-wave method. *Phys. Rev. B* **59**, 1758-1775 (1999).

23 Nørskov, J. K. *et al.* Origin of the overpotential for oxygen reduction at a fuel-cell cathode. *J. Phys. Chem. B* **108**, 17886-17892 (2004).

24 Zhan, C. *et al.* Origins and implications of interfacial capacitance enhancements in C_{60}-modified graphene supercapacitors. *ACS Appl. Mater. Interfaces* **10**, 36860-36865 (2018).

25 Xia, J., Chen, F., Li, J. & Tao, N. Measurement of the quantum capacitance of graphene. *Nat. Nanotechnol.* **4**, 505-509 (2009).

26 Medford, A. J. *et al.* CatMAP: A software package for descriptor-based microkinetic mapping of catalytic trends. *Catal. Lett.* **145**, 794-807 (2015).

27 Hansen, H. A., Varley, J. B., Peterson, A. A. & Nørskov, J. K. Understanding trends in the electrocatalytic activity of metals and enzymes for CO_2 reduction to CO. *J. Phys. Chem. Lett.* **4**, 388-392 (2013).

28 Guo, D. *et al.* Active sites of nitrogen-doped carbon materials for oxygen reduction reaction clarified using model catalysts. *Science* **351**, 361-365 (2016).

29 Sougrati, M. T., Goellner, V., Schuppert, A. K., Stievano, L. & Jaouen, F. Probing active sites in iron-based catalysts for oxygen electro-reduction: A temperature-dependent ^{57}Fe Mössbauer spectroscopy study. *Catal. Today* **262**, 110-120 (2016).

30 Malko, D., Kucernak, A. & Lopes, T. *In situ* electrochemical quantification of active sites in Fe–N/C non-precious metal catalysts. *Nat. Commun.* **7**, 13285 (2016).

31 Dima, G. E., de Vooys, A. C. A. & Koper, M. T. M. Electrocatalytic reduction of nitrate at low concentration on coinage and transition-metal electrodes in acid solutions. *J. Electroanal. Chem.* **554-555**, 15-23 (2003).

32 Colucci, J. A., Foral, M. J. & Langer, S. H. Nitric oxide reduction at noble metal electrodes: A voltammetric study in acid solution. *Electrochim. Acta* **30**, 1675-1685 (1985).

33 Malko, D., Kucernak, A. & Lopes, T. Performance of Fe–N/C oxygen reduction electrocatalysts toward NO_2^-, NO, and NH_2OH electroreduction: From fundamental insights into the active center to a new method for environmental nitrite destruction. *J. Am. Chem. Soc.* **138**, 16056-16068 (2016).

34 Fedoseev, G. *et al.* Efficient surface formation route of interstellar hydroxylamine through NO hydrogenation. II. The multilayer regime in interstellar relevant ices. *J. Chem. Phys.* **137**, 054714 (2012).

35 Bayachou, M., Lin, R., Cho, W. & Farmer, P. J. Electrochemical reduction of NO by myoglobin in surfactant film: Characterization and reactivity of the nitroxyl (NO^-) adduct. *J. Am. Chem. Soc.* **120**, 9888-9893 (1998).

36 Daiber, A. *et al.* Isotope effects and intermediates in the reduction of NO by $P450_{NOR}$. *J. Inorg. Biochem.* **88**, 343-352 (2002).

37 Kuhn, A. T. & Chan, C. Y. pH changes at near-electrode surfaces. *J. Appl. Electrochem.* **13**, 189-207 (1983).

38 Bergel, A., Féron, D. & Mollica, A. Catalysis of oxygen reduction in PEM fuel cell by seawater biofilm. *Electrochem. Commun.* **7**, 900-904 (2005).

39 Auinger, M. *et al.* Near-surface ion distribution and buffer effects during electrochemical reactions. *Phys. Chem. Chem. Phys.* **13**, 16384-16394 (2011).

40 Ryu, J., Wuttig, A. & Surendranath, Y. Quantification of interfacial pH variation at molecular length scales using a concurrent non-Faradaic reaction. *Angew. Chem. Int. Ed.* **57**, 9300-9304 (2018).

41 Yang, K., Kas, R. & Smith, W. A. *In situ* infrared spectroscopy reveals persistent alkalinity near electrode surfaces during CO_2 electroreduction. *J. Am. Chem. Soc.* **141**, 15891-15900 (2019).

42 Monteiro, M. C. O., Jacobse, L., Touzalin, T. & Koper, M. T. M. Mediator-free SECM

for probing the diffusion layer pH with functionalized gold ultramicroelectrodes. *Anal. Chem.* **92**, 2237-2243 (2020).

43 Bedioui, F. *et al.* Design and characterization of chemically modified electrodes with iron(III) porphyrinic-based polymers: Study of their reactivity toward nitrites and nitric oxide in aqueous solution. *Anal. Chim. Acta* **341**, 177-185 (1997).

44 Vilakazi, S. L. & Nyokong, T. Electrocatalytic properties of vitamin B_{12} towards oxidation and reduction of nitric oxide. *Electrochim. Acta* **46**, 453-461 (2000).

45 Immoos, C. E. *et al.* Electrocatalytic reductions of nitrite, nitric oxide, and nitrous oxide by thermophilic cytochrome P450 CYP119 in film-modified electrodes and an analytical comparison of its catalytic activities with myoglobin. *J. Am. Chem. Soc.* **126**, 4934-4942 (2004).

46 Otsuka, K., Sawada, H. & Yamanaka, I. A hydrogen-nitric oxide cell for the synthesis of hydroxylamine. *J. Electrochem. Soc.* **143**, 3491-3497 (1996).

47 Daems, N., Sheng, X., Alvarez-Gallego, Y., Vankelecom, I. F. J. & Pescarmona, P. P. Iron-containing N-doped carbon electrocatalysts for the cogeneration of hydroxylamine and electricity in a H_2–NO fuel cell. *Green Chem.* **18**, 1547-1559 (2016).

48 Sheng, X. *et al.* Carbon-supported iron complexes as electrocatalysts for the cogeneration of hydroxylamine and electricity in a NO-H_2 fuel cell: A combined electrochemical and density functional theory study. *J. Power Sources* **390**, 249-260 (2018).

49 Bathia, M. L. & Watkinson, A. P. Hydroxylamine production by electroreduction of nitric oxide in a trickle bed cell. *Can. J. Chem. Eng.* **57**, 631-637 (1979).
